# Label-Free Evaluation of Advanced Glycation End Products by a Comprehensive Biophysical Analysis

**DOI:** 10.1021/acs.analchem.5c07502

**Published:** 2026-07-25

**Authors:** Ameera K, Darshan Chikkanayakanahalli Mukunda, Subhash Chandra, Shaik Basha, Shimul Biswas, Nirmal Mazumder, Vijendra Prabhu, Manuru Mukhyaprana Prabhu, Jackson Rodrigues, Chih-Wei Hsu, Sheng-Bo Lai, Ajeetkumar Patil, Chia-Yuan Chang, Guan-Yu Zhuo, Krishna Kishore Mahato

**Affiliations:** † Department of Biophysics, Manipal School of Life Sciences, 539679 Manipal Academy of Higher Education, Manipal, Karnataka 576104, India; ‡ Department of Biosciences and Bioengineering, Indian Institute of Technology Dharwad, Dharwad, Karnataka 580011, India; § Department of Biotechnology, Manipal Institute of Technology, Manipal Academy of Higher Education, Manipal, Karnataka 576104, India; ∥ Department of Medicine, Kasturba Medical College, Manipal Academy of Higher Education, Manipal, Karnataka 576104, India; ⊥ Institute of Biophotonics, 390197 National Yang Ming Chiao Tung University, Taipei 112304, Taiwan; # Department of Mechanical Engineering, 34912 National Cheng Kung University, Tainan 70101, Taiwan; ∇ Manipal Institute of Applied Physics, Manipal Academy of Higher Education, Manipal, Karnataka 576104, India

## Abstract

Nonenzymatic glycation is a post-translational modification of proteins, leading to the formation of advanced glycation end products (AGEs) implicated in diabetes, neurodegenerative disorders (NDDs), and aging-related complications. The sensitive and precise detection of protein-bound AGEs is essential for comprehending their pathological implications. Conventional detection methods for AGEs have several drawbacks, such as being expensive and time-consuming, complex sample preparation steps, etc. Further, AGEs’ structural complexity and heterogeneity affect their accurate determination. Therefore, we introduce an autofluorescence-based methodology (LED-induced autofluorescence) for detecting and tracking the formation of AGEs on proteins using a low-power fiber-coupled near-UV LED (340 nm) and a blue LED (430 nm) as excitation sources covering the heterogeneous excitation maxima of multiple AGEs that form on proteins. The device captures the wide range of AGEs-specific autofluorescence signatures without the interference of protein autofluorescence, revealing their heterogeneous composition on proteins. The device demonstrated high sensitivity in detecting standard pentosidine (3.66 pg/μL) at 340 nm excitation, while enabling effective monitoring of the formation and progression of clinically relevant AGEs such as pentosidine, argpyrimidine, vesperlysine (A, B, and C), etc., generated on various structurally distinct glycated proteins in vitro. While noninvasive diabetes risk assessment and diagnosis based on AGEs fluorescence is gaining growing attention, the use of low-power, cost-effective light-emitting diodes (LEDs) as excitation sources offers significant advantages. Their stable power output ensures high reproducibility, enabling a rapid and reliable approach to assessing protein glycation, facilitating the routine monitoring of accumulation of AGEs, and advancing research addressing glycation-related complications in diabetes, aging, and NDDs.

## Graphical abstract

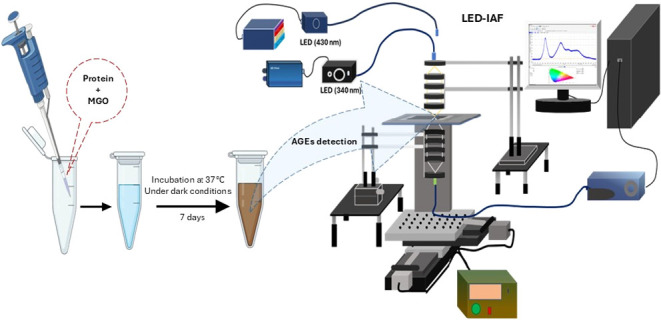

Glycation refers to the nonenzymatic reaction between reducing sugars or their carbonyl derivatives and free amino groups in proteins to form unstable Schiff bases and Amadori products, which continue to generate advanced glycation end products (AGEs).^1,2^ AGEs are associated with oxidative stress, protein aggregation, and dysfunction in cellular and tissue levels and play a crucial role in diabetes, cardiovascular diseases, and neurodegeneration.^3–5^ Understanding glycation and its impact on protein structure and stability is important for the development of therapeutic strategies for the management of AGE-associated disorders.

Methylglyoxal (MGO) is a highly reactive dicarbonyl compound produced as a byproduct of glycolysis via the unregulated breakdown of various triose phosphate (aldose) intermediates (e.g., glyceraldehyde 3-phosphate and dihydroxyacetone phosphate) in glucose catabolism. MGO is constantly produced by the usual metabolic processes and usually detoxified by the glyoxalase system. However, under stressful metabolic conditions, including hyperglycemia, oxidative stress, and metabolic disorders, MGO levels increase significantly in cells, leading to increased glycation of proteins and higher concentrations of AGEs. MGO can rapidly modify proteins because of its high reactivity with arginine (Arg) and lysine (Lys). This leads to the formation of structurally and functionally altered biomolecules-associated diabetic complications such as retinopathy, nephropathy, and neuropathy.^6,7^ MGO directly reacts with amino acid residues, resulting in the formation of AGEs at a considerably faster rate than what occurs with the Maillard reaction. The rate of MGO-mediated glycation mimics carbonyl stress observed in vivo, and therefore, the MGO-mediated glycation pathway is commonly used as an experimental model to characterize structural or functional changes caused by glycation of proteins. This model provides both practical and biologically relevant means of investigating the formation of AGE and their effect on protein stability and aggregation.^8,9^ In this study, MGO was used as the glycation agent to induce in vitro glycation of model proteins. Due to its broad application as a model for investigating structural and biophysical changes related to glycation of proteins, MGO is also widely utilized to evaluate methods developed for AGE detection.^10–14^ Also, protein-bound AGEs can be generated under controlled and reproducible conditions within reasonable timeframes using MGO as a glycation agent.

Even though conventional methods (Table S1), including high-performance liquid chromatography (HPLC), fluorescence assays, liquid chromatography–mass spectrometry (LC–MS), and immunodetection techniques, are used for AGEs detection, these methods usually require complicated sample preparation, expensive reagents, long processing steps, and sophisticated instruments.^1,15^ Hence, a fast, efficient, and label-free reliable detection method is needed to improve the glycation research. AGEs are stable, accumulate over time, and exhibit autofluorescence, making fluorescence spectroscopy a sensitive tool for detecting long-term metabolic stress and protein modifications.^16–18^ Noninvasive fluorescence-based approaches enable large-scale screening, disease severity assessment, and early intervention using a single excitation source (∼370 nm).^17,19–22^ Integrating dual-LED technology further enhances this approach by offering highly reproducible diagnostic platforms.^23,24^ Though UV–C and UV–B light used to induce autofluorescence in glycated proteins,^25,18^ the in vivo application is limited by phototoxicity and shallow tissue penetration.^26–28^ In contrast, low-power near-UV (∼340 nm) and blue (∼430 nm) LEDs more effectively align with the excitation characteristics of AGEs compared to 370 nm excitation alone, which can overlook key fluorophores such as argpyrimidine and vesperlysine C. Based on this, we developed an LED-induced autofluorescence (LED-IAF) spectroscopy system incorporating 340 nm and 430 nm LEDs for label-free detection of AGE-associated fluorescence. This dual-wavelength approach enhances detection coverage while also providing improved tissue penetration and reduced cytotoxicity, making it more suitable for in vivo applications.^23,24^ Further, the device coupled the LED-IAF with a high-resolution CCD spectrometer for comprehensive spectral characterization, enabling improved signal-to-noise and accurate identification of AGE-associated fluorescence.

To validate the LED-IAF, in vitro glycation of three standard proteins, HSA, RNase A, and lysozyme, was carried out under optimized conditions.^13^ The performance of the LED-IAF was cross-validated with an indirect enzyme-linked immunosorbent assay (ELISA) and LC–MS. Further, the fluorescamine assay, 8-anilinonaphthalene-1-sulfonic acid (ANS) and thioflavin T (ThT) assays, Fourier transform infrared (FTIR) spectroscopy, dynamic light scattering (DLS), X-ray powder diffraction (XRPD), and fluorescence lifetime imaging microscopy (FLIM) were used for an in-depth understanding of glycation-induced alteration probed by LED-IAF.

## Experimental Section

### Materials

Human serum albumin, ribonuclease A, lysozyme, bovine serum albumin, and l-lysine were procured from Himedia Laboratories (Mumbai, India). 8-anilino-1-naphthalene-sulfonic acid (ANS), methylglyoxal (40% aqueous solution), thioflavin T (ThT), Tween 20, anti-AGE antibody (AB9890), and mouse antigoat IgG peroxidase conjugated (AP186P) were procured from Sigma-Aldrich (St. Louis, MO, USA). Pentosidine (trifluoroacetate salt) was procured from Cayman Chemicals (USA).

### Instrumentation

The LED-IAF device was designed (Figure 1) and developed in-house. The setup, portable and comprising two different fiber-coupled LEDs emitting at 340 nm (model no. FCS-0340–001, Mightex Systems, California, USA) and 430 nm (model no. M430F1, Thorlabs, New Jersey, USA), was used as an excitation source. A fused silica biconvex lens (part no. LB4879, Thorlabs, New Jersey, USA) (L1) of focal length (f) 35 mm (positioned at focal distance from the fiber delivery end) couples the LED light from the fiber, making it parallel through (a) a 343 nm bandpass filter (Part No. FBH343–10, Thorlabs, New Jersey, USA) to specifically select 340 nm excitation or (b) a 430 nm bandpass filter (Part No. FBH430–10, Thorlabs, New Jersey, USA) to select 430 nm excitation. This filtered light of a specific wavelength is then focused onto the sample by using another biconvex lens (*f* = 35 mm) (L2). The lenses and filters are arranged inside the stackable flip filter mount (part no. FP-FCH-25, Holmarc Opto-Mechatronics Ltd., India) so that one filter can be moved from the light path when the other is in use. The setup also consists of a motorized XY translational stage (model no. LMS 100200–1, Holmarc Opto-Mechatronics Ltd., India), which is attached to a customized angle bracket with quartz plate mount housing the sample containing a cuvette, precisely positioning the point of excitation. The XY micromovement of the translation stage was remotely monitored using dedicated software through a computer. In the collection geometry, the setup consists of two biconvex lenses (L3 and L4) to couple the signal from the samples under study into the collection fiber for detection through filters: a 360 nm long-pass edge filter (Part No. LP02–360RU-25, Semrock Inc., New York, USA) and a 450 nm long-pass filter (Part No. FELH0450, Thorlabs, New Jersey, USA), eliminating respective excitations (either 340 or 430 nm) from the collected fluorescence. An optical fiber collects the filtered autofluorescence from the sample and sends it to the Compact CCD Spectrometer (QE65000, Ocean Optics, Inc., USA) for detection. The CCD spectrometer is directly connected to a computer, and the spectral signatures are acquired using OceanView software.

**Figure 1:**
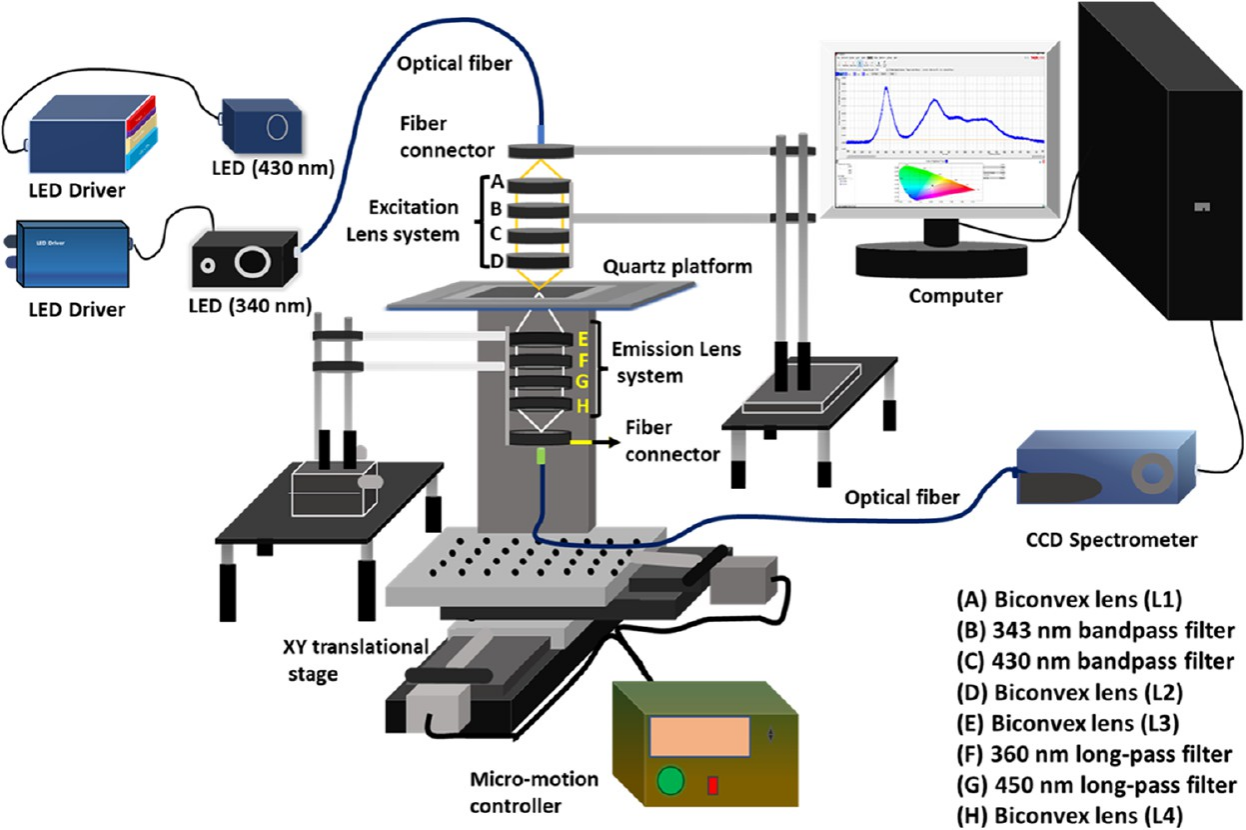
Block diagram of the in-house developed LED-IAF instrument. The LED-IAF system integrates two fiber-coupled LEDs (340 and 430 nm) with respective bandpass filters for selective excitation of AGEs. Fluorescence from the sample, aligned on an XY translational stage, is collected through optical lenses and long-pass filters, and delivered to a CCD spectrometer via an optical fiber. The spectral output is recorded and analyzed using specific software.

### Sample Preparation

25 μM protein samples (HSA, lysozyme, and RNase A) were prepared in phosphate buffer (pH 7.4). Varying MGO concentrations (0.2, 0.4, 0.6, 0.8, 1, 2, 4, 6, 8, and 10 mM) were used to treat the protein samples and kept for incubation under dark conditions at 37 °C for 7 days. Various concentrations of glycated-HSA and pentosidine were used to test the sensitivity of the LED-IAF setup. Solution samples ranging from 1 nM to 1 mM concentrations were prepared in phosphate buffer (pH 7.4).^13,29^

### LED-IAF Spectroscopy

After incubation, the autofluorescence spectra of the samples were recorded using the in-house-built LED-IAF device. The emission spectrum between 350 and 700 nm was recorded at 340 nm excitation. For 430 nm excitation, a spectral range between 440 and 800 nm was recorded. Deep-UV-IAF spectroscopy was performed using a pre-existing setup designed to study intrinsic protein fluorescence. The emission spectrum between 300 and 700 nm was recorded at an excitation of 285 nm.^13^

### Fluorescamine Assay

Glycated protein samples were mixed with 100 μL of 100 mM Na_2_HPO_4_ buffer (pH 7.4), 45 μL of distilled water, and 50 μL of fluorescamine reagent (1 mM in acetonitrile) in a 96-well plate. The reaction mixtures were incubated for 10 min at room temperature in the dark. Fluorescence was measured using a plate reader (Varioskan ALF, Thermo Fisher Scientific) at excitation and emission wavelengths of 345 and 450 nm, respectively. Freshly prepared HSA and phosphate-buffered saline PBS were used as controls for nonglycated protein and background fluorescence, respectively.^30,31^ The percentage of the free amino group was calculated using eq ([Disp-formula eq1]).

1 $$\mathrm{free}\;\mathrm{amino}\;\mathrm{group}\;\left(\%\right)=\frac{\mathrm{fluorescence}\;\mathrm{emission}\;\mathrm{of}\;\mathrm{sample}}{\mathrm{fluorescence}\;\mathrm{emission}\;\mathrm{of}\;\mathrm{control}}\times 100$$

### Indirect ELISA

An indirect ELISA was carried out to evaluate the formation of AGEs in glycated protein samples independently. The following were coated onto 96-well plates: native HSA and MGO-treated HSA samples, MGO solutions (0.2, 1, and 10 mM), and standard pentosidine (5 nM, 500 nM, and 5 μM). The samples were diluted in carbonate–bicarbonate coating buffer (pH 9.6), added to wells, and incubated overnight at 4 °C to facilitate their adsorption to the wells. The wells were washed 3 times with PBS containing 0.05% Tween 20 (PBST) and then blocked with 3% bovine serum albumin (BSA) in PBS for 1 h at room temperature. Following blocking, a solution of the anti-AGE primary antibody (AB9890, Merck) was diluted in blocking buffer and incubated for 2 h at room temperature with the previously coated sample. After washing with PBST, the HRP-conjugated secondary antibody (AP186P, Merck) was added and incubated for 1 h. The wells were then washed thoroughly, and the tetramethylbenzidine (TMB) substrate was added to develop color. The reaction was terminated with sulfuric acid, and the absorbance of each well was measured at 450 nm using a multimode microplate reader (Varioskan ALF, Thermo Fisher Scientific).^15,32^ The blank value from the wells was subtracted from each of the absorbance measurements before analysis. Each experiment was conducted in triplicate.

### Spectral Quantification with Non-Negative Classical Least Squares (NN-CLS)

NN-CLS spectral decomposition was performed using the reference fluorophore spectra of pentosidine and vesperlysine to estimate their relative spectral contribution within the measured spectra of glycated HSA at a given concentration of MGO at 340 nm excitation. This approach enabled assessment of relative changes in individual AGE-specific fluorescence signals.^33^ The measured fluorescence spectrum of glycated HSA was modeled as a combination of the reference spectra of pentosidine and vesperlysine A/B, with a relatively low residual value representing the unexplained spectral contributions from crossline and other arginine-derived AGEs, as explained by eq ([Disp-formula eq2]).

2 X=CS+E

In the equation, *X* represents the measured spectrum, *S* is the matrix of reference spectra, *C* is the vector of contribution coefficients, and *E* represents the residual component. The coefficients were estimated by solving a non-negative least-squares optimization problem using the lsqnonneg algorithm in MATLAB, ensuring physically meaningful (non-negative) solutions. The coefficients representing the relative contributions of pentosidine and vesperlysine A/B were estimated using the non-negative least-squares algorithm (lsqnonneg, MATLAB), which restricts the coefficients to non-negative values (≥0) [eq ([Disp-formula eq3])]. The resulting non-negative coefficients were then used to compute the relative fractional area of each pure component spectrum over the total integrated spectral area.

3 $$\min{||X-\mathrm{SC}\left|\right|}^{2}\;\mathrm{subject}\;\mathrm{to}\;C\geq 0$$

The obtained coefficients were subsequently normalized to yield the relative contributions of each component. A threshold value of 2% was applied to the normalized coefficients to determine the presence or absence of the individual component. Model performance was evaluated by calculating the reconstruction error between the original and fitted spectra, along with a visual comparison of spectral agreement. All data processing and analysis were carried out in MATLAB 2026a software (MathWorks, USA).

### Liquid Chromatography–Mass Spectrometry (LC–MS)

Protein glycation was analyzed using LC–MS (Agilent AdvanceBio 6545XT LC/Q-TOF system, Agilent Technologies, USA). Samples were injected into a reverse-phase C8 column (2.1 × 150 mm, 3.6 μM). Samples were eluted using water, acetonitrile mix with 0.1% formic acid as the mobile phase, with a flow rate of 0.3 mL/min. The column temperature was maintained at 45 °C. Mass spectrometric analysis was performed over an *m*/*z* range of 500–3000. The total ion chromatogram (TIC) was recorded, and data acquisition was followed by spectral deconvolution and peak assignment. The baseline of all the spectra was corrected, followed by smoothing.

### ANS Fluorescence Assay

The ANS fluorescence assay was used to determine the modified protein structure. For the test, 500 μM ANS dye was incubated with the sample for 30 min at room temperature. After incubation, a Multimode Microplate Reader (Spark,Tecan) was used to record fluorescence emission spectra from 450 to 700 nm at an excitation wavelength of 380 nm.^34^

### Thioflavin T Fluorescence Assay

To determine the protein aggregation, the ThT assay was employed. The protein solution (100 μL) was incubated with 900 μL of 10 mM ThT reagent at 37 °C. Fluorescence emission was measured from 450 to 600 nm at an excitation of 400 nm using a microplate reader (Spark, Tecan). Fresh HSA solution, dye, and buffer were used as controls.^35^

### FTIR Spectroscopy

The KBr (potassium bromide) pellet method of the FT/IR-4X FTIR spectrometer (JASCO, Ltd., Tokyo, Japan) fitted with an MCT (mercury cadmium and telluride) detector was used to measure the lyophilized samples. To obtain a spectrum with a significant signal-to-noise ratio, we removed the background spectrum. The spectra were recorded within the 600–4000 cm^–1^ spectral range.^14^

### Dynamic Light Scattering (DLS)

The glycation experiments were performed as previously stated. Excess unreacted MGO was removed by centrifugal filtration using the Millipore ultrafiltration tube (3 kDa) (Millipore, MA, USA) for 30 min at 5000 rpm. The hydrodynamic diameter (Dh) and particle size distribution (polydispersity index, PDI) of filtered (0.22 μm) protein samples were assessed by DLS utilizing a Nano-ZS type laser particle size analyzer (Zeta Nano-ZS, Malvern Instruments). Every experiment was carried out at 25 °C. The average value was calculated after each sample was measured three times in parallel. Water, which had a refractive index of 1.33, served as the dispersion, and the substance was protein, which had a refractive index of 1.45.^36,37^

### X-ray Powder Diffraction (XRPD) Analysis

The XRPD patterns of lyophilized protein samples (native and glycated) were recorded using an Ultima IV X-ray diffractometer (Rigaku, Tokyo, Japan) featuring crossbeam optics (CBO) technology. The data was collected over a range of 2θ from 5 to 90° while maintaining optimum temperature conditions. The raw data was processed for background correction and baseline normalization before analysis.^38^

### Fluorescence Lifetime Imaging Microscopy (FLIM)

The integrated Two-Photon FLIM system comprised of an ultrafast laser (Chameleon Ultra II, Coherent), a pulse width of 140 fs, a repetition rate of 80 MHz, and the wavelength adjustable in the range of 680–1080 nm. The laser pulse train was monitored by a silicon-pin photodiode (TDA 200, PicoQuant) for pulse timing. The beam scanning was performed using an XY galvanometer scanner (6215H, Cambridge Technology) into an inverted microscope (Eclipse TE2000-U, Nikon) with a 20×/0.8 NA air objective (UPLXAPO20X, Olympus) mounted on a piezo scanner (PD72Z4CAQ, Physik Instrumente). A motorized 3-axis stage (H117E1N4, Prior Scientific) enabled large-area imaging.^39^ The protein samples, native HSA and HSA treated with 10 mM MGO, were prepared in solution using phosphate-buffered saline of 7.2 pH. FLIM measurements were performed using the excitation wavelengths at 750 and 840 nm, and the corresponding images were acquired separately. The photon emission at visible wavelengths were collected for lifetime analysis. The excitation power of 750 and 840 nm applied on the sample was 5 and 10 mW, respectively, to produce two-photon fluorescence. Scanning was conducted over a 200 μm field of view, with fluorescence images recorded (256 × 256 pixels), and corresponding FLIM data was acquired at 128 × 128 pixels. Data acquisition produced a fluorescence intensity image, a FLIM lifetime map, a decay curve obtained by 3 × 3 pixel binning centered at the region of interest for fitting, and a histogram of fluorescence lifetime (ns) versus pixel count.

## Results and Discussion

### Sensitivity of the Device for Free Pentosidine Detection by 340 nm LED-IAF

Pentosidine is a cross-linking (Lys→Lys), fluorescent (λ_em_∼385 nm) AGE and a potential biomarker for diabetes control, renal failure, and aging.^40,41^ Its accumulation causes protein cross-linking (e.g., in elastin and collagen), contributing to tissue stiffness, vascular complications, and bone fragility.^42–44^ In the current study, pentosidine was employed as a standard fluorescent AGE to evaluate the sensitivity and analytical performance of the LED-IAF platform. The calibration curve of pentosidine served as a reference for LOD and linearity of the device rather than a direct measure of total AGEs content in glycated protein samples. Pentosidine produced an emission peak at ∼385 nm (Figure 2a), in line with fluorescence emission patterns previously documented in the literature.^13,30^ The device demonstrated an LOD of 10 nM, reflecting the notable sensitivity. Results also showed that fluorescence intensities were linear as a function of pentosidine concentrations (Figure 2b), with an *R* ^2^ value of 0.9979 attributed to an exceptional power stability (Figure S1) of the LED excitation source. Based on the estimated volume of sample illuminated by the LED source (∼6.28 μL), calculated from the cylindrical excitation area created by the 2 mm diameter light spot and 2 mm optical path length, the overall practical limit for absolute detection of pentosidine was found to be 3.66 picograms/μL, falling within a range relevant to physiological levels of pentosidine reported in biological systems.^45,46^

**Figure 2:**
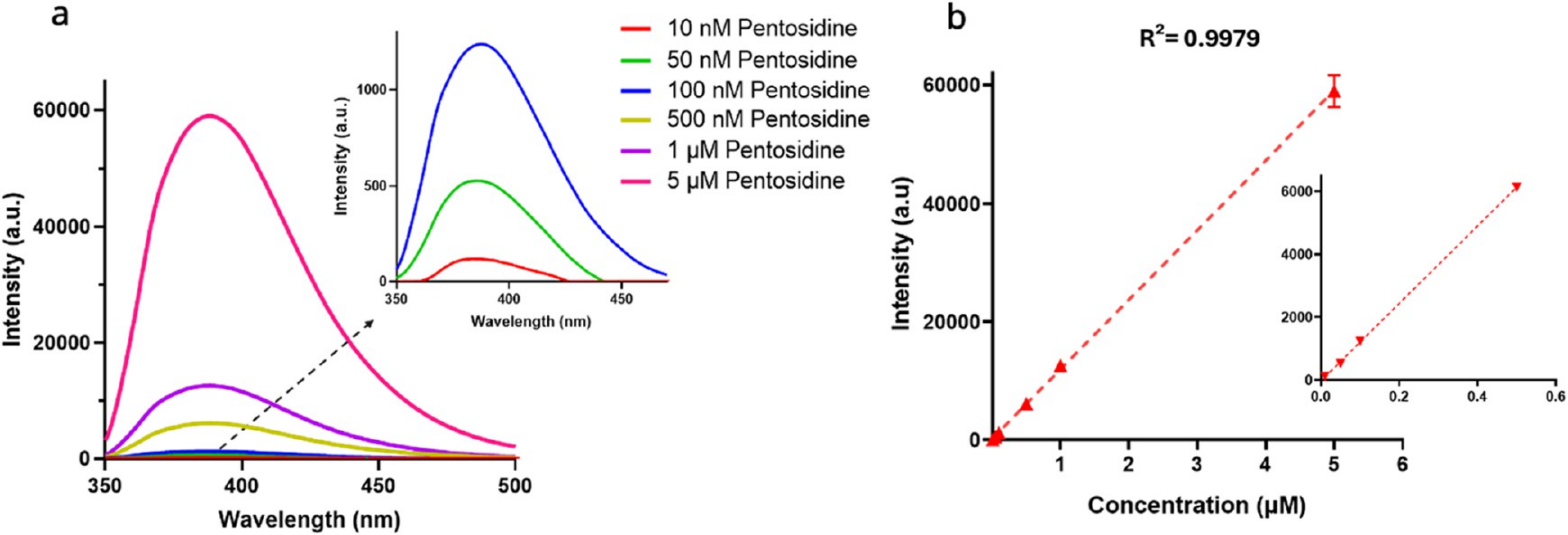
Detection of free pentosidine by 340 nm LED-IAF: (a) The autofluorescence spectra of various concentrations (10 nM–μM) of pentosidine at 340 nm LED excitation with an integration time of 10s. The spectral patterns of lower concentrations (10, 50, and 100 nM) are shown in the figure inset. All measurements were performed in triplicate. (b) Linearity plot showing the relationship between fluorescence intensity and pentosidine concentration.

### Sensitivity of the Device for Detecting Protein-Bound AGEs

In vitro glycated HSA, treated with 0.2 mM MGO, characterized by 4.78 ± 1.96% of glycation, corresponding to the modification of 4–5 primary amino residues, was used to evaluate the sensitivity of the device. Despite the minimal modification, distinct AGE-associated fluorescence signals were observed at 1 μM protein concentration, underscoring the device’s ability to detect early-stage glycation (Figure 3). In contrast, under identical excitation conditions, 1 μM native HSA (unmodified) did not produce a detectable AGE-specific fluorescence (Figures S2 and S3), confirming that the detected signal in glycated HSA originated specifically from AGEs. However, at its higher concentrations (10 μM), the native HSA also showed AGE-specific fluorescence (Figures S2 and S3). Consistent with this observation, ELISA measurements (Figure 9) revealed the presence of AGEs in the unmodified HSA. This clearly suggests that the commercially sourced HSA contains a basal level of naturally occurring AGEs.

**Figure 3:**
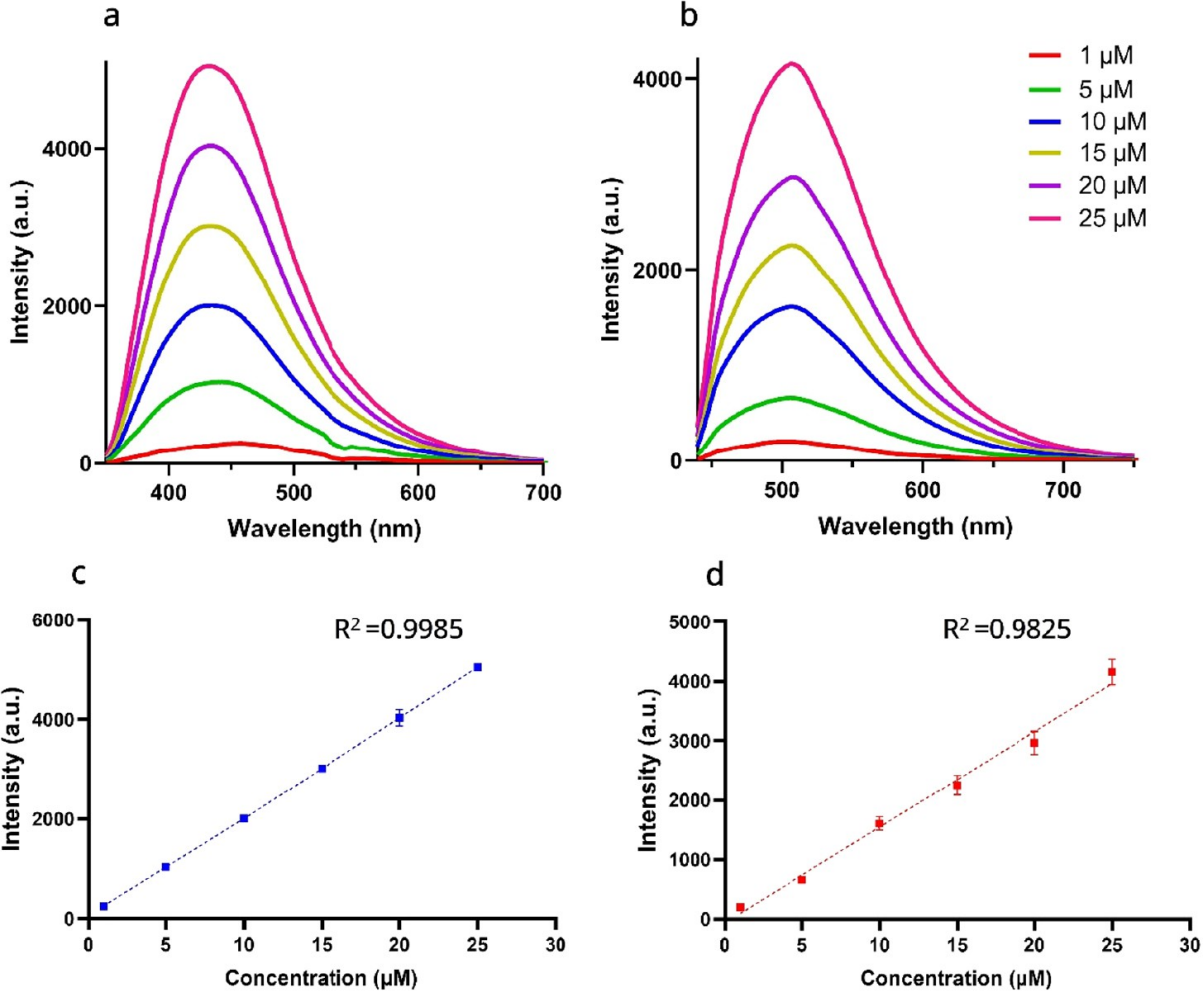
LED-IAF spectra of glycated HSA: The autofluorescence spectra of various concentrations (1–25 μM) of glycated HSA, (a) in the spectral range 350–700 at 340 nm LED excitation with an integration time of 5s and (b) in the spectral range 440–750 at 430 nm LED excitation with an integration time of 1s. (c and d) The corresponding linearity plot showing the relationship between fluorescence intensity and glycated HSA concentration.

### LED-IAF of Glycated Proteins

The LED-IAF spectra (350–700 nm) of glycated proteins exhibited an increase in fluorescence intensity compared to native proteins at 340 nm excitation, confirming the formation of AGEs. Native HSA (unmodified) showed slight but measurable AGE-specific fluorescence (Figure 4), reinforcing the sensitivity of LED-IAF in detecting AGE-related modifications. The presence of AGE-specific fluorescence in the unmodified HSA is likely due to pre-existing endogenous AGEs formed during the physiological lifespan of albumin in vivo. Owing to its prolonged circulating half-life (∼21 days) and multiple lysine and arginine residues, commercially available plasma-derived HSA contains a measurable level of fluorescent AGEs. Further, the in vitro glycated HSA under 340 nm excitation showed a gradual increase in fluorescence intensity with an emission maximum at ∼400 nm, as a function of increasing MGO concentrations until 0.8 mM. Interestingly, at 1 mM MGO treatment, the emission maximum of glycated HSA shifted to 447 nm, broadening the emission spectra and decreasing the fluorescence intensity, as a result of a diverse population of AGEs formed at higher glycation concentration levels (Figure 4a,b).^13,47^ The higher proportion of AGEs that emit at shorter wavelengths (e.g., pentosidine: λ_em_ ≈ 380–390 nm; argpyrimidine: λ_em_ ≈ 395–400 nm; vesperlysine C: λ_em_ ≈ 405 nm) formed at lower concentrations of MGO are responsible for low wavelength emission.^13^ In contrast, at higher concentrations of MGO, there are elevated levels of AGEs that emit at relatively longer wavelength (vesperlysine A and B: λ_em_ ≈ 445 nm; crossline: λ_em_ ≈ 480–490 nm and other Arg-derived AGEs: λ_em_ ≈ 520 nm). Although AGEs with emission maxima at longer wavelengths are not efficiently excited at 340 nm, the FRET (Förster resonance energy transfer) from shorter-wavelength-emitting AGEs formed on the same protein molecule leads to the excitation of AGEs such as vesperlysine A, vesperlysine B, and crossline whose excitation spectrum overlap with the emission spectrum of pentosidine (Figure 2a), argpyrimidine, and vesperlysine C, resulting in a red shift in the emission maxima, spectral broadening, and a lower net fluorescence intensity, as shown in Figure 4.^48–50^ The concentration-dependent red shift in the emission maxima of MGO-modified proteins suggest a progressive alteration in the composition of fluorescent AGEs generated during glycation. Different AGEs exhibit distinct emission characteristics (Table 1). Upon excitation at 340 nm, in vitro glycated RNase A demonstrated a red shift in emission maxima from 410 to 440 nm, a gradual increase in fluorescence intensity, and a broadened spectral range with increasing MGO concentrations (Figure 4c,d). A continuous increase in fluorescence intensity with increasing MGO concentrations, despite the red shift and spectral broadening, suggests that AGEs like vesperlysine (A, B, and C) are the predominant adducts formed in glycated RNase A.^51^ The spectral broadening observed indicates the simultaneous formation of a heterogeneous population of AGEs that have overlapping emission properties, including both short- and long-wavelength-emitting species.^1^ However, at 340 nm excitation, the fluorescence intensity of in vitro glycated lysozyme increased up to 0.8 (mM) MGO concentration, after which it started decreasing and exhibited a broader spectral pattern. The reduction in intensity at higher MGO levels may be due to the structural changes, increased cross-linking, or energy transfer among accumulated AGEs.^13^ The spectral broadening indicates the formation of a diverse mixture of AGEs with overlapping emission (Figure 4e,f).

**Figure 4:**
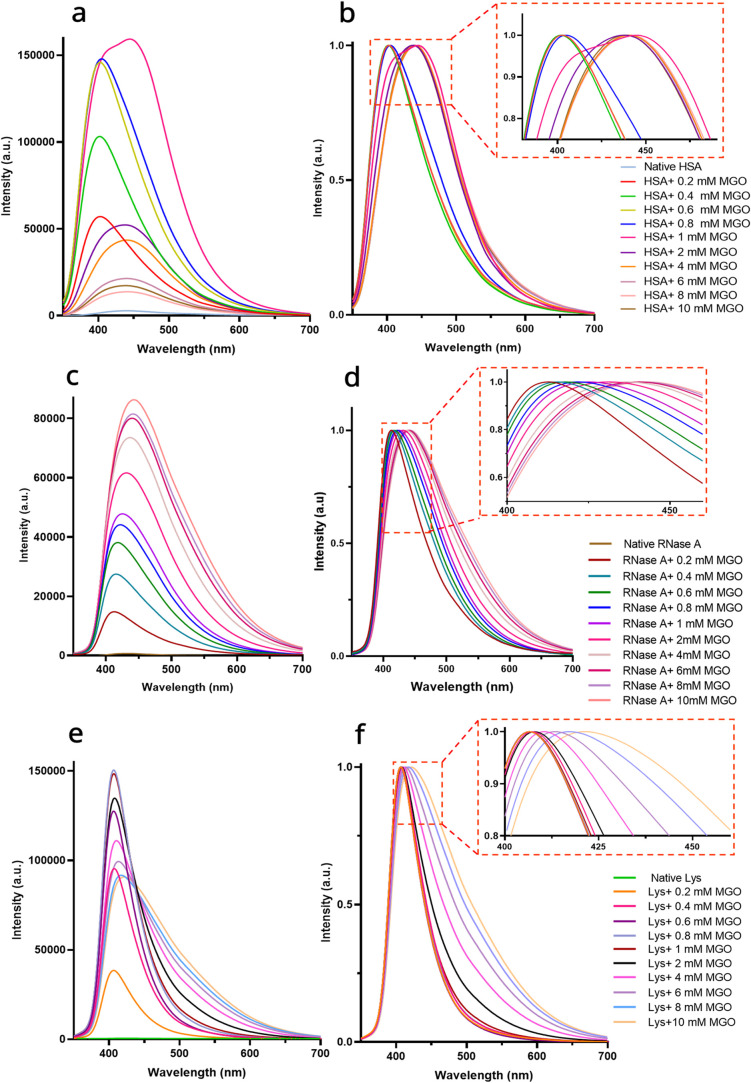
LED-IAF spectra at 340 nm excitation: Fluorescence spectral patterns of native and MGO-modified (0.2–10 mM) proteins upon excitation with a 340 nm LED. Emission spectra were recorded in the 350–700 nm range. To enable comparison of spectral shapes across different MGO concentrations, a unity-based normalization was performed by scaling each spectrum to its maximum intensity (peak intensity = 1). (a) LED-IAF spectra of HSA and (b) the corresponding normalized spectra, (c) LED-IAF spectra of RNase A and (d) the corresponding normalized spectra, and (e) LED-IAF spectra of lysozyme and (f) the corresponding normalized spectra. All measurements were performed in triplicate.

**Table 1:** Excitation and Emission Wavelengths of Different Fluorescent AGEs

**AGE**	**excitation wavelength**	**emission wavelength**	**refs**
pentosidine	320–340 nm	380–385 nm	^52^
vesperlysine A and B	370 nm	440 nm	^48,18^
vesperlysine C	345 nm	405 nm	^48,18^
argpyrimidine	320 nm	395 nm	^53^
crossline	420 nm	480 nm	^13^

At an excitation wavelength of 430 nm, the fluorescence intensity increased with increased concentration of MGO, suggesting AGE accumulation. Although there was an evident increase in intensity, the emission maxima were largely unchanged for all protein samples, with maximum fluorescence emission around 510–520 nm, consistent with arginine-derived AGEs.^13^ In the case of HSA and RNase A, at a high concentration of MGO, the emission spectra exhibited distinguishable shoulder peaks at ∼480 nm, suggesting the formation of crossline.^14^ However, lysozyme showed a contrasting behavior. At lower MGO concentrations, a 480 nm shoulder was present, but as the MGO concentration increased, the emission spectra became narrower, lacking the more pronounced 480 nm shoulder present at lower concentrations (Figure 5). Overall, the three glycated proteins under study, HSA, RNase A, and lysozyme, exhibited distinct fluorescence emission patterns upon excitation at 340 and 430 nm, reflecting differences in their glycation behavior based on the lysine–arginine composition. These differences may arise from variations in amino acid composition, glycation sites, and protein conformational stability.

**Figure 5:**
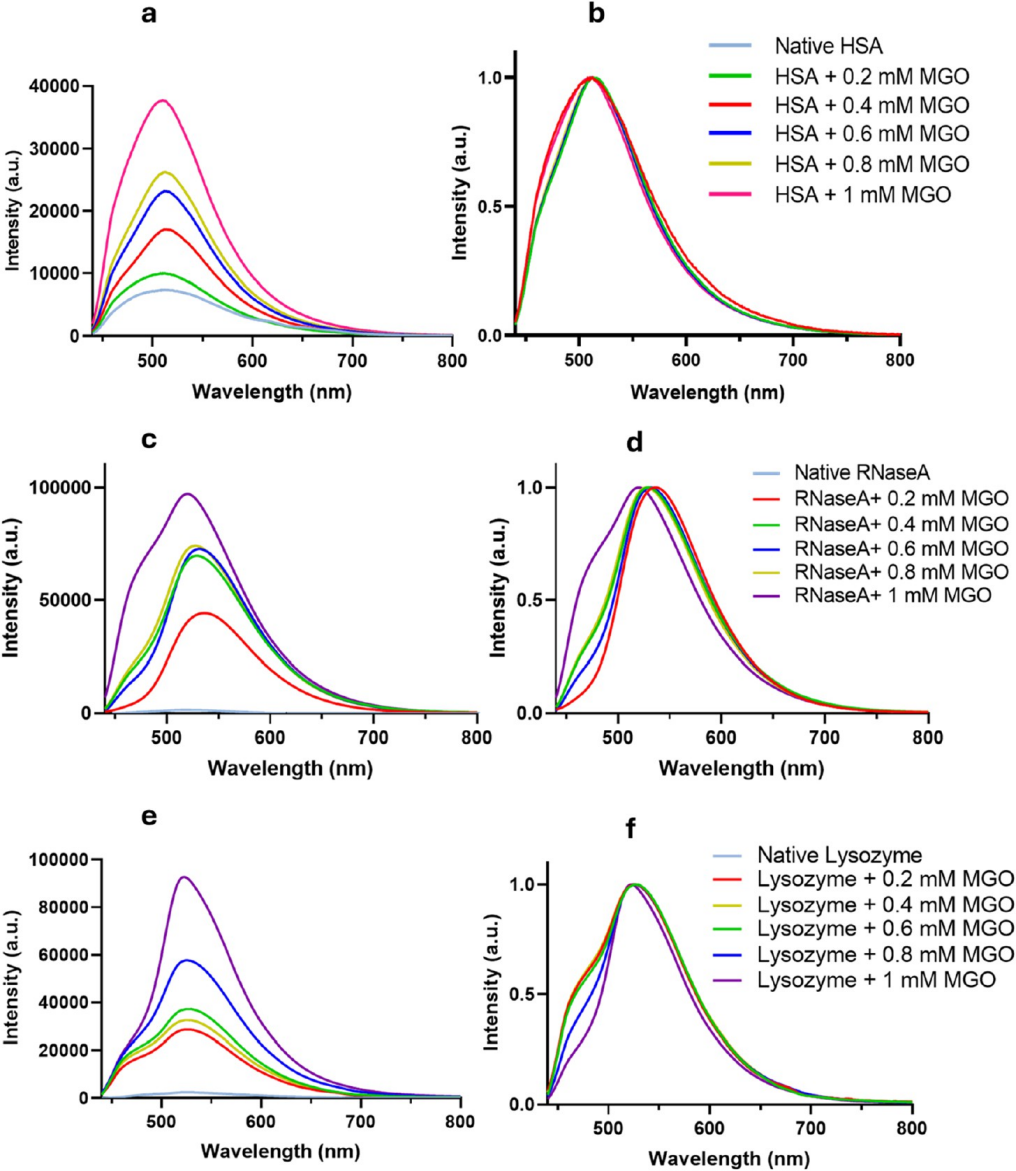
LED-IAF spectra at 430 nm excitation: Fluorescence spectral patterns of native and MGO-modified (0.2–1 mM) proteins upon excitation with a 430 nm LED. Emission spectra were recorded in the 440–800 nm range. To enable comparison of spectral shapes across different MGO concentrations, unit normalization was performed by scaling each spectrum to its maximum intensity (peak intensity = 1). (a) LED-IAF spectra of HSA and (b) the corresponding normalized spectra, (c) LED-IAF spectra of RNase A and (d) the corresponding normalized spectra, and (e) LED-IAF spectra of lysozyme and (f) the corresponding normalized spectra. All measurements were performed in triplicate.

### Spectral Quantification with Non-Negative Classical Least Squares (NN-CLS)

Quantification of protein-bound AGEs by fluorescence spectroscopy remains challenging due to the heterogeneous nature of AGE fluorophores, their overlapping emission profiles, and energy transfer between them.^54,55^ Furthermore, fluorescence intensity is influenced by the local protein environment, quenching effects, and differences in fluorophore quantum yields, which alter the relationship between signal intensity and AGE concentration.^18,56,47^ The spectral shift in glycated proteins arises from changes in AGE composition. NN-CLS analysis of glycated HSA showed that the fluorescence of pentosidine and vesperlysine fluorophores accounts for a major portion of the observed experimental fluorescence (Figure 6). Pentosidine fluorescence predominated at low MGO concentrations, while the contribution of vesperlysine fluorescence increased with higher MGO levels. The standard fluorescence spectra of pentosidine and vesperlysine are shown in Figure S4. Though the emission maxima were centered at ∼400 nm (λ_ex_ = 340 nm) at lower MGO concentrations, a significant contribution of vesperlysine A/B fluorescence was revealed by NN-CLS, as shown in Figure 6b–e. Interestingly, emission maxima at ∼441 nm component in the case of unmodified HSA suggest that vesperlysine contributes more substantially to the endogenous fluorescence of unmodified HSA than pentosidine.^13,30^ Furthermore, the ability of a two-component model to reproduce the experimental spectra with high fidelity suggests that pentosidine and vesperlysine-like fluorophores represent the principal spectral determinants of MGO-induced fluorescence in proteins. The nonzero lack of fit (LOF) values (Figure 6) suggest that, in addition to pentosidine and vesperlysine, other fluorescent AGEs contribute to the overall spectra of MGO-modified proteins. This observation is consistent with the complex chemistry of the MGO-induced glycation, which generates a diverse population of AGEs^13,14^ with overlapping characteristics. Overall, these findings demonstrate that spectral unmixing can provide insight into the evolving AGE composition during glycation.

**Figure 6:**
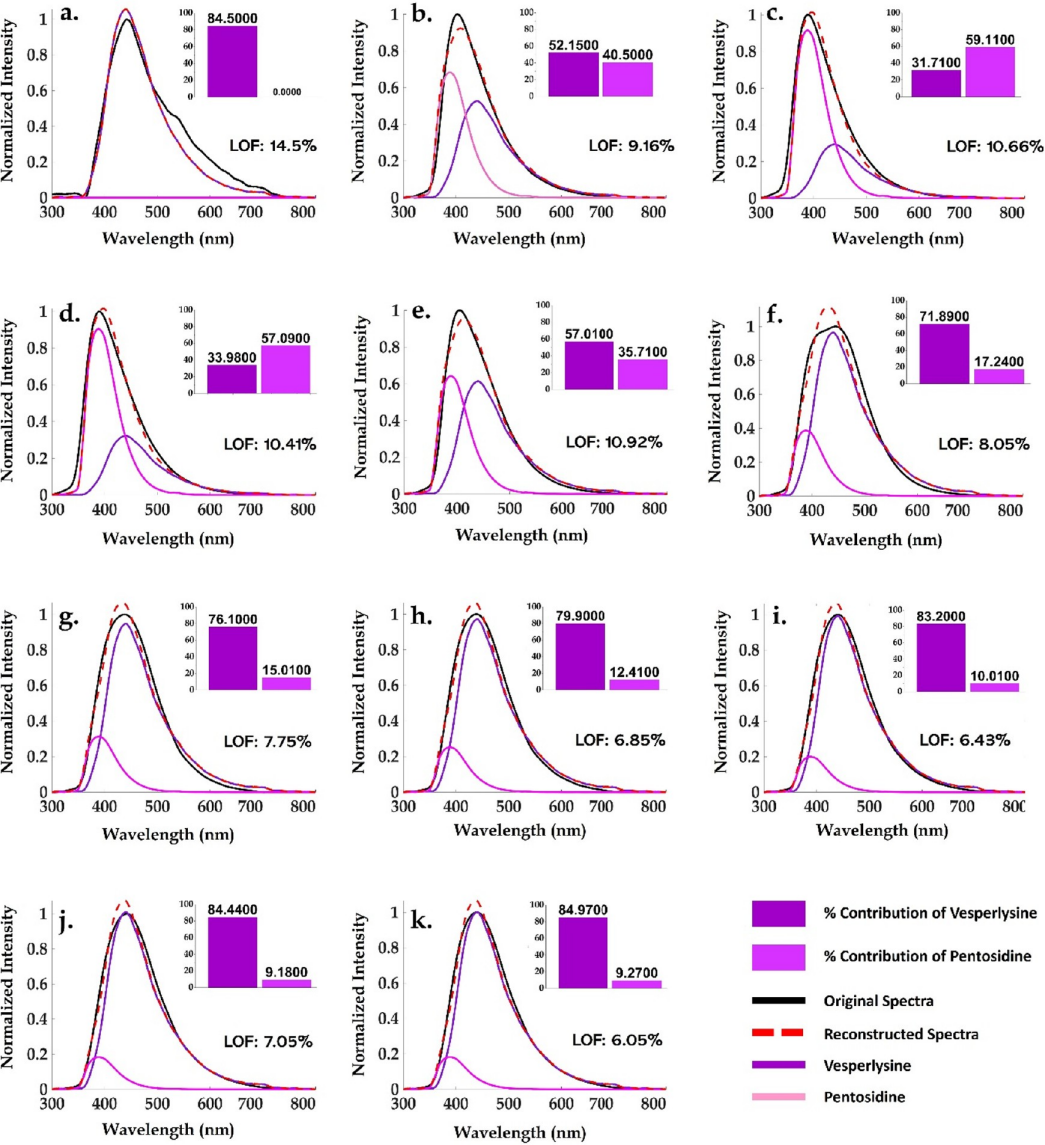
NN-CLS reconstruction of fluorescence spectra of native and MGO-glycated HSA using pentosidine (light purple) and vesperlysine (dark purple) as reference fluorophores. Panels represent (a) native HSA and HSA treated with (b) 0.2 mM, (c) 0.4 mM, (d) 0.6 mM, (e) 0.8 mM, (f) 1 mM, (g) 2 mM, (h) 4 mM, (i) 6 mM, (j) 8 mM, and (k) 10 mM MGO.

### Fluorescamine Assay

The site-specific modification of proteins by MGO was demonstrated by the fluorescamine assay, which demonstrated a significant reduction in free primary amines, indicating lysine and arginine. The preferential targeting arises from the high nucleophilicity of the ε-amino group of lysine and the guanidino group of arginine.^57,58^ Fluorescamine is a nonfluorescent reagent that reacts with free primary amino acids to yield highly fluorescent compounds, making it a sensitive probe for accessible amine groups in proteins.^31^ Modification of amino groups by MGO progressively reduces the availability of primary amines for fluorescamine binding, resulting in a corresponding decline in fluorescence intensity (Figure 7). This decrease reflects the extent of MGO-mediated modification of lysine and arginine residues. At lower MGO concentrations (0.2 mM), a minimal reduction in free amino groups was observed (95.21 ± 1.96% free amino groups), indicating limited modification (4.78 ± 1.96%). In contrast, at higher MGO concentrations (10 mM), a substantial loss of free amino groups was evident, with only 56.25 ± 7.88% remaining.^59,60^

**Figure 7:**
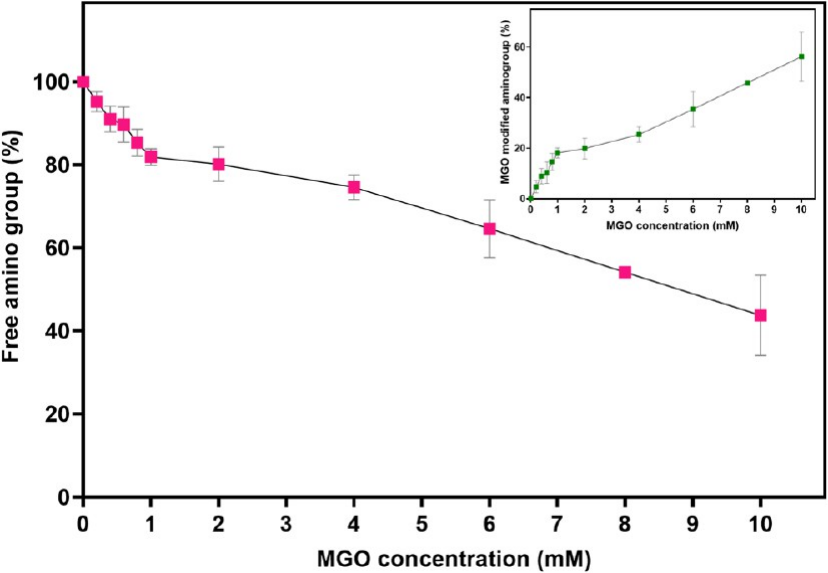
Free amino groups in glycated HSA. Percentage of free amino groups in MGO-modified (0.2 mM-10 mM) HSA, indicating progressive glycation; measurements were performed in triplicate. Inset: Percentage of MGO-modified amino groups.

### LED-IAF of Elastin

Elastin, a long-lived extracellular matrix protein with minimal turnover, was investigated to assess the applicability of the LED-IAF method for detecting AGEs in biologically relevant systems (Figure S5). Due to its prolonged lifetime, elastin is highly susceptible to nonenzymatic glycation and AGE accumulation. Upon excitation at 340 nm, an emission maximum at ∼424 nm was observed, while excitation at 430 nm produced emission in the ∼480–502 nm range. The emission maxima of elastin at both excitations (340 and 430) matched the emission maxima of AGE-specific fluorescence recorded for standard proteins at 340 and 430 nm. Standard elastin exhibits intrinsic AGE-related fluorescence due to cumulative nonenzymatic glycation occurring in vivo prior to isolation.^61,62^ Its long biological half-life and susceptibility to glycoxidative modifications result in the retention of endogenous fluorescent AGE species. Consequently, commercially available elastin inherently displays AGE-associated autofluorescence. Similarly, the absence of protein turnover brings about the accumulation of AGEs in a specialized protein called crystallin present in the eye lens. Therefore, detection of AGEs in the eye lens is critically important because these modifications accumulate with age and contribute to protein cross-linking, pigmentation, and loss of lens transparency, ultimately leading to cataract formation.^63^

### Deep-UV-IAF of Glycated Proteins

To further explore the fluorescence spectral patterns at 340 and 430 nm excitation, additional fluorescence spectroscopy was performed at 285 nm (UV–C) excitation using a pre-existing setup designed to study intrinsic protein fluorescence.^13^ This wavelength specifically excites tryptophan and tyrosine residues, typically exhibiting emission around 320–350 nm in nonglycated proteins. However, in glycated HSA, the fluorescence spectra displayed the expected intrinsic emission and exhibited additional peaks around 380, 440, 480, and 520 nm. The appearance of these AGE-associated peaks at 285 nm excitation suggests the appearance of FRET from tryptophan and tyrosine residues to the protein-bound AGEs. It can be demonstrated by comparing the 340 and 430 nm spectra with the 285 nm excitation spectra (Figure 8) that the broad fluorescence at both 340 and 430 nm excitation is a consequence of a combination of emission from multiple AGE species. The validation with 285 nm excitation and the examination of the intrinsic fluorescence of HSA show that, upon glycation, there is a significant change in the intrinsic protein fluorescence, which further supports the AGE formation, involved in modifying the protein structure.^13,14^

**Figure 8:**
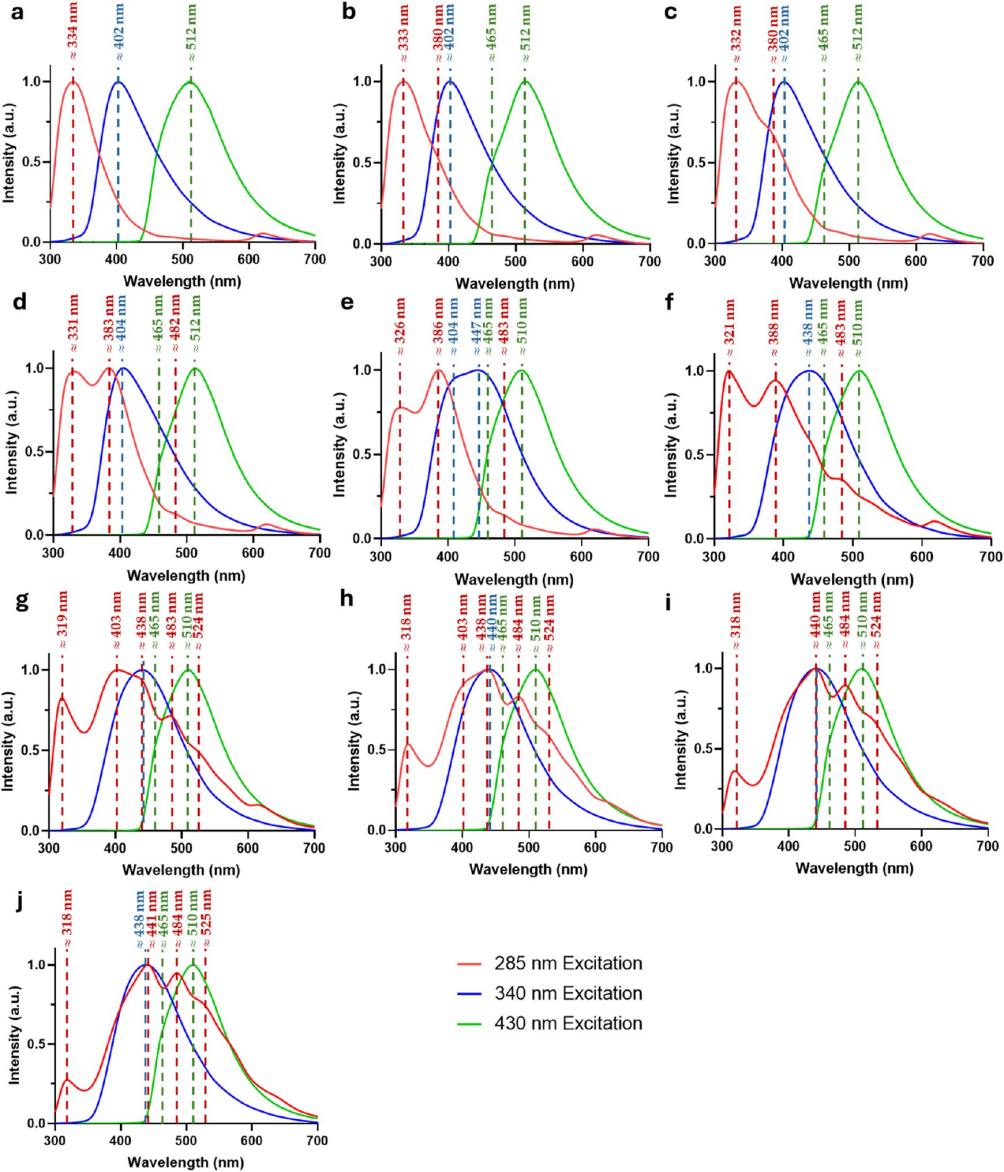
Comparison of LED-IAF spectra at multiple excitation wavelengths. LED-IAF spectra of MGO-modified HSA recorded at excitation wavelengths of 285, 340, and 430 nm. Spectral changes were observed with increasing MGO concentrations: (a) 0.2 mM, (b) 0.4 mM, (c) 0.6 mM, (d) 0.8 mM, (e) 1 mM, (f) 2 mM, (g) 4 mM, (h) 6 mM, (i) 8 mM, and (j) 10 mM. These spectra reflect the progressive accumulation of fluorescent AGEs and conformational changes in HSA with increasing glycation stress. All measurements were performed in triplicate, and the spectra were unit-normalized to enable comparison of spectral profiles. The dotted lines indicate the fluorescence peaks (red −285 nm, blue −340 nm, and green −430 nm excitation).

### Indirect ELISA

Indirect ELISA was performed to independently verify MGO-induced AGE formation in protein samples. An anti-AGE immunoreactivity was observed with increasing MGO concentration. Native HSA exhibited significantly elevated immunoreactivity (Figure 9). The highest response was recorded for HSA glycated with 10 mM MGO, indicating the substantial accumulation of AGEs under elevated glycation conditions. In contrast, free MGO and pentosidine standards produced absorbance values close to those of the blank level. This clearly suggests the specificity of the polyclonal anti-AGE antibody toward protein-bound AGEs. An important finding of this study is that a measurable AGE-associated fluorescence was observed in unmodified HSA (Figure 4a), and this observation was validated by ELISA-based AGE detection. This clearly indicates that the LED-IAF device is not just limited to detecting experimentally induced AGEs but is sensitive enough to detect endogenous AGE modifications in physiologically glycation-prone proteins such as albumin, hemoglobin, collagen, elastin, and eye lens crystalline.

**Figure 9:**
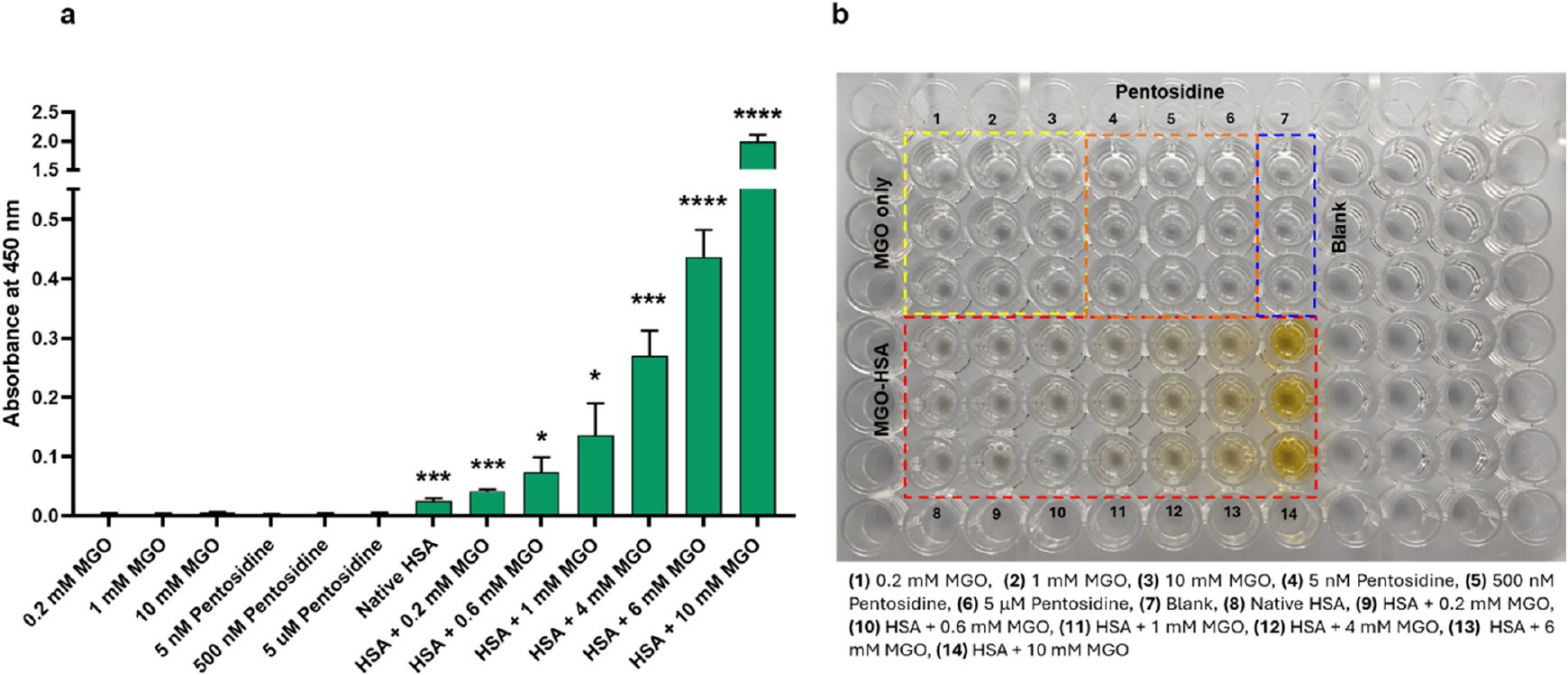
ELISA validation of AGE formation in MGO-glycated HSA. (a) Blank-corrected absorbance at 450 nm for native HSA, MGO-glycated HSA, free MGO, and pentosidine standards (b) Representative ELISA plate image. Data are presented as mean ± SD (*n* = 3). Significance was determined by one-way ANOVA followed by Dunnett’s T3 multiple-comparison post-hoc test (*p ≤ 0.05; ** *p* ≤ 0.01; *** *p* ≤ 0.001; **** *p* ≤ 0.0001).

### Mass Spectrometry (ESI-MS)

Electrospray ionization mass spectrometry (ESI-MS) was employed to evaluate the intact mass of native RNase A and MGO-treated RNase A.^64^ The ESI mass spectrum of native RNase A displayed a well-defined charge envelope corresponding to a molecular mass of ∼13.683.09 kDa (Figure 10a,b). Upon MGO treatment, a systematic shift of the charge envelope toward higher *m*/*z* values was observed, yielding a deconvoluted mass of ∼13.827.10 kDa (dominating peak) with a net charge increase by +144 Da (Figure 10c,d). Importantly, the deconvoluted spectrum (Figure 10d) of MGO-treated RNase A did not exhibit a single discrete peak but rather a distribution of species spanning ∼13,683–14,231 Da, indicating the presence of a heterogeneous population of glycated protein forms. This mass distribution suggests progressive and multisite modification of RNase A by MGO. To systematically interpret these mass shifts, the observed deconvoluted masses were tabulated and correlated with known MGO-derived modifications (Table 2).^65^ Analysis of the tabulated data revealed characteristic mass increments of approximately +72 Da, +54 Da, and +58 Da, which are consistent with the previous reports of MGO-modified proteins.^65,66^ The presence of a prominent +144 Da mass shift indicates the formation of doubly modified RNase A species, while higher mass increments (up to +548 Da) reflect multisite glycation and accumulation of multiple adducts. The stepwise increase in mass observed across the deconvoluted spectrum forms a characteristic “mass ladder,” representing the successive addition of MGO-derived adducts. The observed heterogeneity is consistent with the presence of multiple reactive nucleophilic sites within RNase A, particularly arginine (4 residues) and lysine (10 residues) residues. However, the dominance of +72 Da-based increments and the significant +144 Da species indicate that arginine residues are the primary targets of MGO modification, in agreement with previous reports. Overall, the mass spectrometric analysis demonstrates that MGO induces extensive structural modification of RNase A, resulting in a heterogeneous population of glycated species. The combined evidence from raw mass spectra, deconvoluted mass analysis, and systematic interpretation of mass increments provides strong confirmation of progressive, multisite glycation and the formation of AGEs under the experimental conditions.

**Figure 10:**
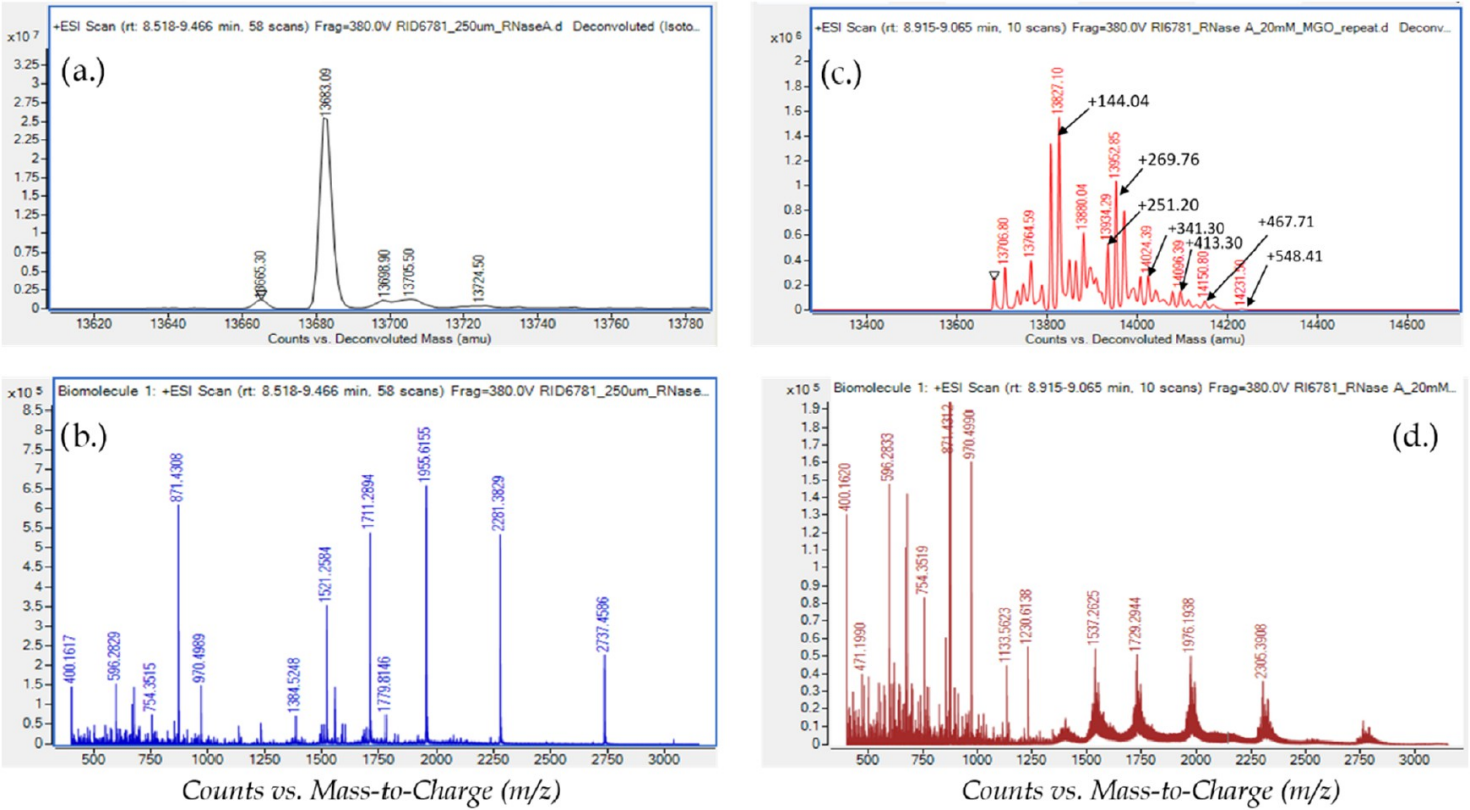
Electrospray ionization mass spectrometry (ESI-MS) analysis of native and MGO-modified RNase A. (a) Deconvoluted spectrum of native RNase A confirming a molecular mass of ∼13,683 Da. (b) Raw mass spectrum of native RNase A showing a well-defined charge state distribution (*z* = 5–9). (c) Deconvoluted spectrum of MGO-modified RNase A showing multiple mass species with a dominant peak at ∼13,827 Da (+144 Da), indicating progressive multisite glycation. (d) Raw mass spectrum of MGO-treated RNase A exhibiting a broadened charge envelope with a shift toward higher *m*/*z* values.

**Table 2:** Summary of Deconvoluted Mass Peaks Obtained for MGO-Modified RNase A, Including Observed Masses, Corresponding Mass Shifts (Δ*m*), and Proposed Modification Combinations

**observed mass**	**Δmass (Da)**	**mass combination (Da)**
13683.09	0	0
13827.1	144.01	72 + 72 = 144.04
13934.29	251.20	72 + 72 + 54 + 54 = 252
13952.85	269.76	72 + 72 + 72 + 54 = 270
14024.4	341.30	72 + 72 + 72 + 72 + 54 = 342
14096.4	413.30	72 + 72 + 72 + 72 + 72 + 54 = 413
14150.8	467.71	72 + 72 + 72 + 72 + 72 + 54 + 54 = 468
14231.5	548.41	72 + 72 + 72 + 72 + 72 + 72+ 58 + 58 = 548

### ANS Fluorescence Assay

The fluorescence assay using ANS was used to evaluate changes in surface hydrophobicity due to structural changes mediated by glycation. The ANS dye binds to exposed hydrophobic sites on proteins, and it can be used as a sensitive indicator of glycation-mediated changes to the structure of a protein.^67^ For HSA, the glycated form had a significantly lower ANS fluorescence intensity compared to the control glycated protein (Figure 11a), which suggests that HSA likely underwent unfolding and buried the hydrophobic regions.^68^ However, RNase A and lysozyme had a slight increase in hydrophobicity (Figure 11b,c), which may be attributed to their compact structure and lack of extensive unfolding. ANS fluorescence measurements indicate an altered surface hydrophobicity in glycated proteins, suggesting partial unfolding and conformational rearrangement. These changes are consistent with the spectral broadening observed in LED-IAF measurements, indicating that variations in the microenvironmental polarity contribute to the modulation of AGE-associated fluorescence. The results further confirm surface hydrophobicity changes associated with conformational expansion and aggregation, as reported in glycation studies.^13,69,70^

**Figure 11:**
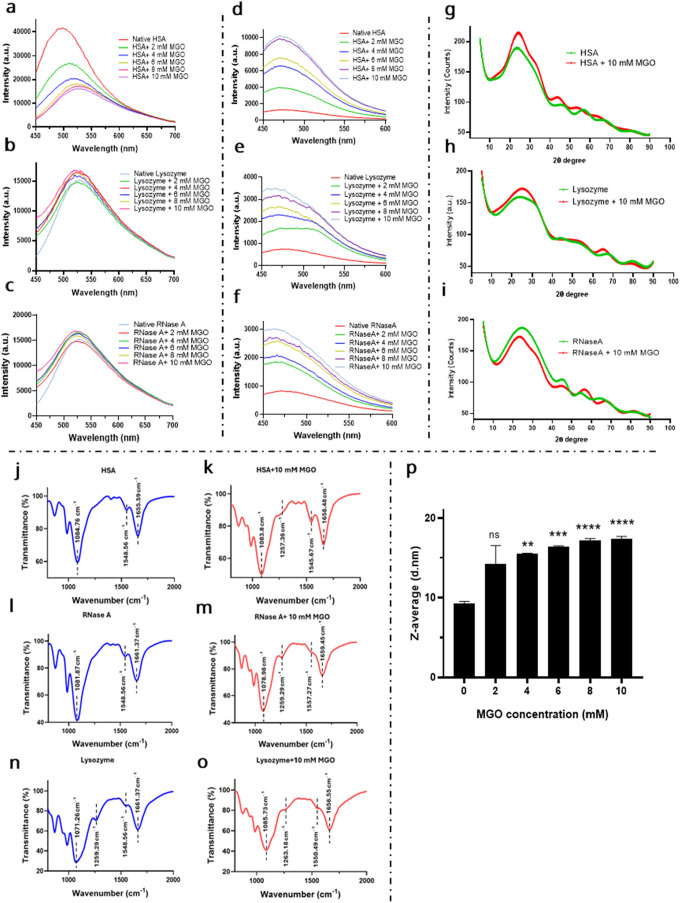
Complementary biophysical and spectroscopic analyses of glycation-induced modifications in proteins. (a–c) ANS fluorescence spectra of native and MGO-modified proteins: HSA, lysozyme, and RNase A, showing changes in surface hydrophobicity. (d–f) ThT fluorescence spectra of native and MGO-modified proteins: HSA, lysozyme, and RNase A, indicating amyloid-like structural features upon glycation. (g–i) XRD patterns of native and MGO-glycated proteins, highlighting alterations in crystallinity and structural organization. (j–o) FTIR spectra of native and MGO-glycated proteins (HSA, RNase A, lysozyme) showing glycation-induced changes in secondary structure, particularly in the amide I and amide II regions; dotted lines mark characteristic peak positions. (p) Z-average of native and MGO-modified (2–10 mM) HSA, presented as mean ± SEM (*n* = 3); significance determined by one-way ANOVA with Tamhane’s T2 post-hoc test (**** *p* ≤ 0.0001; *** *p* ≤ 0.001; ** *p* ≤ 0.01).

### ThT Fluorescence Assay

Protein aggregation was then further studied using the ThT assay. ThT is a benzothiazole dye that has specificity for intermolecular β-sheet structures typically associated with protein aggregates or amyloids.^71^ As such, the ThT assay revealed a far greater ThT fluorescence intensity in the glycated proteins, confirming β-sheet-rich aggregate development^67^ (Figure 11d–f). Specifically, the increase in ThT fluorescence intensity for glycated HSA compared with glycated RNase A and lysozyme suggests that glycation alters the structure of HSA to a larger extent and facilitates greater internal protein aggregation.^29^ The greater susceptibility of glycation, larger protein size, and ability to form larger aggregates with more β-sheet structures may explain HSA’s higher aggregate formation. The increase in LED-IAF fluorescence intensity with increasing MGO concentration is consistent with the enhancement in ThT fluorescence, indicating the formation of β-sheet-rich aggregates upon glycation. This suggests that, in addition to AGE formation, aggregation-associated structural rearrangements contribute to the observed fluorescence changes, particularly spectral broadening and red shifts.^72^

### FTIR Spectroscopy

FTIR spectra of native and MGO-glycated proteins revealed notable changes in characteristic vibrational bands, indicating structural modifications upon glycation (Figure 11j–o). In native HSA, prominent peaks were observed at 1084.76 cm^–1^, 1548.56 cm^–1^ (amide II), and 1655.59 cm^–1^ (amide I). Glycated HSA showed a slight shift in these bands to 1083.8, 1545.67, and 1658.48 cm^–1^, along with a new peak at 1257.36 cm^–1^, suggesting the introduction of new functional groups, likely from glycation adducts.^14^

For RNase A, native samples showed peaks at 1081.87, 1548.56, and 1661.37 cm^–1^, while the glycated form exhibited bands at 1078.98, 1259.29, 1557.27, and 1659.45 cm^–1^. The emergence of a new peak at 1259.29 cm^–1^ and shifts in the amide I and II regions further support glycation-induced conformational changes. These spectral shifts collectively indicate modifications in the protein backbone structure and the formation of MGO-derived glycation products.

Native lysozyme exhibited peaks at 1071.26, 1259.29, 1548.56, and 1661.37 cm^–1^. Upon glycation, these shifted to 1085.73, 1263.18, 1550.49, and 1656.55 cm^–1^, reflecting alterations in the secondary structure and possible formation of advanced glycation end products. The observed shifts in the amide I and II regions of glycated proteins, along with the emergence of a new band in the 1250–1300 cm^–1^ range, indicate significant alterations in the secondary structure and the formation of new chemical groups associated with glycation. FTIR analysis further reveals changes in hydrogen bonding patterns, reflecting conformational perturbations of the protein backbone. These structural modifications provide a basis for the variations observed in LED-IAF spectra, particularly the shifts in emission maxima arising from the structural changes in the fluorophores.^14,73^

### Dynamic Light Scattering

DLS measurements were performed to evaluate the hydrodynamic size distribution of the protein samples upon glycation with MGO at increasing concentrations. The *Z*-average hydrodynamic diameter of the native protein was relatively low and consistent with either a monomeric state or a minimally aggregated state. Upon increasing concentrations of MGO, there was an increase in the *Z*-average, suggesting the formation of protein aggregates (Figure 11p). DLS measurements revealed a progressive increase in hydrodynamic diameter with increasing glycation, indicating the formation of higher-order protein assemblies. This increase in particle size suggests enhanced intermolecular interactions and cross-linking induced by MGO. These structural changes provide evidence for glycation-driven modifications that influence the observed fluorescence behavior.^74^

### X-ray Powder Diffraction Analysis

XRPD analysis demonstrated notable changes in the structure between native and glycated protein samples (Figure 11g–i). For HSA, the native diffraction showed broad halo-like features at 20°, indicating an amorphous structure, while glycated HSA showed a higher intensity profile, indicating ordered molecular arrangements. RNase A showed prolific changes due to glycation, shifting from an amorphous native pattern to lower-intensity diffraction, indicating increased molecular disorder or heterogeneity. Lysozyme showed minimal structural changes upon glycation, with both native and glycated forms displaying broad amorphous patterns; a slight 20–30° increase in 2θ peak intensity suggested minor early-stage conformational changes or aggregation.^75,76^ XRPD measurements indicated changes in the solid-state structural organization of glycated proteins, particularly at higher MGO concentrations. These observations reflect alterations in overall packing and an increase in structural disorder rather than direct detection of specific glycation adducts. Given that XRPD is inherently less sensitive to surface-level chemical modifications in amorphous systems, the technique is employed here to capture bulk structural perturbations induced by glycation under conditions where such changes become appreciable. These results provide complementary evidence of glycation-associated structural reorganization.^77^

### Fluorescence Lifetime Imaging Microscopy

To further confirm the occurrence of glycation-derived fluorophores and complement the LED-IAF measurements, FLIM was performed with native and glycated HSA samples. FLIM reveals the heterogeneity between individual AGEs via detecting multiple fluorescence lifetime components, thereby providing complementary and spatially resolved information to the spectral data obtained from LED-IAF.^78^ FLIM measurements were performed using a two-photon excitation source at 750 and 840 nm, corresponding to twice the one-photon excitation maxima of AGEs. For MGO-treated HSA excited at 750 nm (Figure 12a–d), the fluorescence intensity image revealed heterogeneous signal distribution, indicating variation in fluorophore density. The FLIM map showed lifetime domains of ∼0.8–1.4 ns, with the histogram displaying two main populations near ∼0.82 and ∼1.2 ns, suggesting at least two distinct fluorescent species. At 840 nm excitation (Figure 12e–h), similar heterogeneity in fluorophore distribution was observed, with lifetime domains of ∼1.25–2.25 ns and histogram peaks near ∼1.3 and ∼1.6 ns. In both cases, decay curves from 3 × 3 pixel regions of interest showed a good fit between experimental data and fitted exponential models, confirming the reliability of the lifetime estimations. The native HSA was also subjected to the same protocol, and there was no absorption from the sample; as a result, the FLIM image could not be derived (Figure S6). Varied fluorescence lifetimes observed in glycated HSA samples are due to the presence of heterogeneous populations of AGEs within the protein matrix. Different fluorescent AGEs have distinct excitation and emission characteristics. At two-photon excitation with 750 nm, AGEs like vesperlysine C (λ_ex_ ≈ 345 nm) vesperlysine A and B (λ_ex_ ≈ 380 nm) may preferentially get excited, whereas two-photon excitation with 840 nm can selectively excite crossline- (λ_ex_ ≈ 420 nm) and arginine-derived AGEs (λ_ex_ ≈ 460 nm).^13,48,53,52^ This wavelength-dependent excitation leads to varied emission maxima and lifetime distributions (0.82 and 1.2 ns for 750 excitation; 1.3 and 1.6 ns for 840 nm excitation), highlighting chemical and structural diversity among the AGEs. This heterogeneity is consistent with the results of our LED-IAF study utilizing multiexcitation/emission settings to represent subtle yet different signatures of spectral shape variation of these AGE species. The native HSA was also subjected to the FLIM measurements at the same excitations. However, we could not derive an FLIM image, as there was no absorption by the sample. To provide a reference framework, we considered results from a study conducted by Mukunda et al. They demonstrated that native HSA has significantly shorter lifetime (4.79 × 10^–11^ at λ_ex/em_ 345 nm/385 nm; 1.67 × 10^–10^ at λ_ex/em_ 345 nm/400 nm; 1.44 × 10^–10^ at λ_ex/em_ 405 nm/445 nm) and weaker intrinsic emission compared to glycated forms having longer lifetime (1.70 × 10^–9^ at λ_ex/em_ 345 nm/385 nm; 2.31 × 10^–9^ at λ_ex/em_ 345 nm/400 nm; 8.95 × 10^–10^ at λ_ex/em_ 405 nm/445 nm), often approaching background levels under similar excitation conditions.^13^

**Figure 12:**
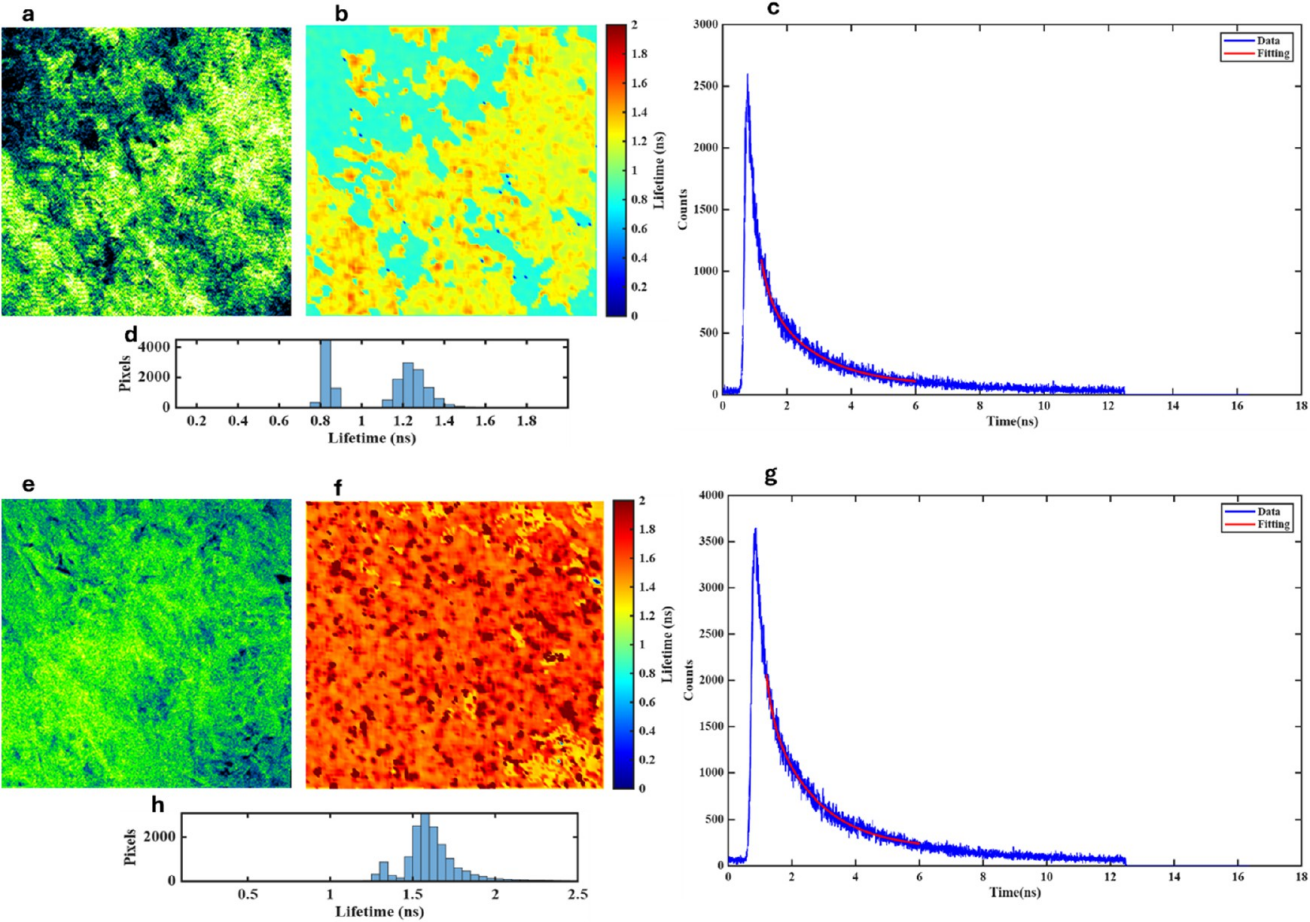
Two-photon FLIM analysis of glycated HSA. Fluorescence intensity images, FLIM lifetime maps, decay curves with fitted models, and lifetime histograms were acquired at 750 nm (a–d) and 840 nm (e–h) excitation. Lifetime analysis (3 × 3 pixel binning at the region of interest) revealed two primary lifetime populations at ∼0.82 ns and ∼1.2 ns for 750 nm and ∼1.3 ns and ∼1.6 ns for 840 nm, corresponding to distinct fluorophore environments within the sample.

## Conclusion

The study established dual-LED-induced autofluorescence (LED-IAF) spectroscopy as a sensitive, label-free method for detecting and tracking AGEs in glycated proteins. The detectable baseline AGE fluorescence in unmodified HSA highlights the high sensitivity of the LED-IAF in detecting the protein-bound fluorescent AGEs. A concentration-dependent increase in fluorescence intensity, along with shifts in emission maxima, enabled tracking progressive formation of heterogeneous AGEs, while mass spectrometry confirmed the accumulation of glycation adducts on multiple sites of the protein. The site-specific modification was corroborated by the fluorescamine assay, which demonstrated a significant reduction in free primary amino groups, indicating lysine and arginine modification. Notably, FLIM highlighted the presence of heterogeneous AGE populations within the protein matrix. Coupling the LED-IAF system with a high-resolution CCD detector improved the resolution of overlapping AGE spectra, overcoming a key limitation of conventional approaches used in noninvasive AGE detection where the spectral congestion often compromises specificity. Given the growing importance of noninvasive AGE assessment in evaluating hyperglycemia and predicting diabetic complications, the current approach shows strong potential for translation into a sensitive, portable, point-of-care diagnostic device for measuring AGEs in vivo as well as ex vivo. Further, the AGE detection serves not only as a marker for glycation but also as an indicator of progressive protein structural remodeling. Complementary techniques consistently revealed a transition toward aggregation-prone states, including increased β-sheet content, enhanced surface hydrophobicity, and conformational expansion. Taken together, the study underscores the protein-bound AGE profiling along with the mechanistic link between AGE formation and protein misfolding-relevant diseases.

## Supplementary Material



## Data Availability

The data sets generated and analyzed during the current study are available from the corresponding author upon request.

## Notes

Additional experimental details, including LED power stability, device sensitivity, pentosidine and vesperlysine spectral reference, AGE-specific fluorescence of elastin, the fluorescence decay profile of native HSA, and a comparison table of AGE detection techniques (PDF)
