# Supplementary material for: Label-Free Evaluation of Advanced Glycation End Products by a Comprehensive Biophysical Analysis
Source: Anal Chem. 2026 Jul 25;98(31):22647–65. doi: 10.1021/acs.analchem.5c07502 (PMC13470982; doi:10.1021/acs.analchem.5c07502)
Supplement: Supplementary file 1 [file ac5c07502_si_001.pdf]

SUPPORTING INFORMATION

Label Free Evaluation of Advanced Glycation End Products by Comprehensive Biophysical Analysis

Ameera K[1], Darshan Chikkanayakanahalli Mukunda[1, 2], Subhash Chandra[1], Shaik Basha[1], Shimul Biswas[1], Nirmal Mazumder[1], Vijendra Prabhu[3], Manuru Mukhyaprana Prabhu[4], Jackson Rodrigues[5], Chih-Wei Hsu[6], Sheng-Bo Lai[6], Ajeetkumar Patil[7], Chia-Yuan Chang[6], Guan-Yu Zhuo[5], Krishna Kishore Mahato[1 *]

1. Department of Biophysics, Manipal School of Life Sciences, Manipal Academy of Higher Education, Manipal – 576104, Karnataka, India.
2. Department of Biosciences and Bioengineering, Indian Institute of Technology Dharwad, Dharwad-580011, Karnataka, India.
3. Department of Biotechnology, Manipal Institute of Technology, Manipal Academy of Higher Education, Manipal – 576104, Karnataka, India.
4. Department of Medicine, Kasturba Medical College, Manipal Academy of Higher Education, Manipal – 576104, Karnataka, India.
5. Institute of Biophotonics, National Yang Ming Chiao Tung University, Taipei 112304, Taiwan
6. Department of Mechanical Engineering, National Cheng Kung University, Tainan 70101, Taiwan
7. Manipal Institute of Applied Physics, Manipal Academy of Higher Education, Manipal – 576104, Karnataka, India.

[*]Corresponding author: mahato.kk@manipal.edu, kkmahato@gmail.com

Table of Contents

1. Power stability of LED excitation sources (340 nm & 430 nm) S2
2. Sensitivity of the LED-IAF device S2
3. Vesperlysine Reference Spectrum For NN-CLS S3
4. AGEs specific fluorescence of Elastin S3
5. Fluorescence decay curve of Native HSA S4
6. Comparison of AGE detection techniques S5
7. References S7

1. Power stability of LED excitation sources (340 nm & 430 nm)

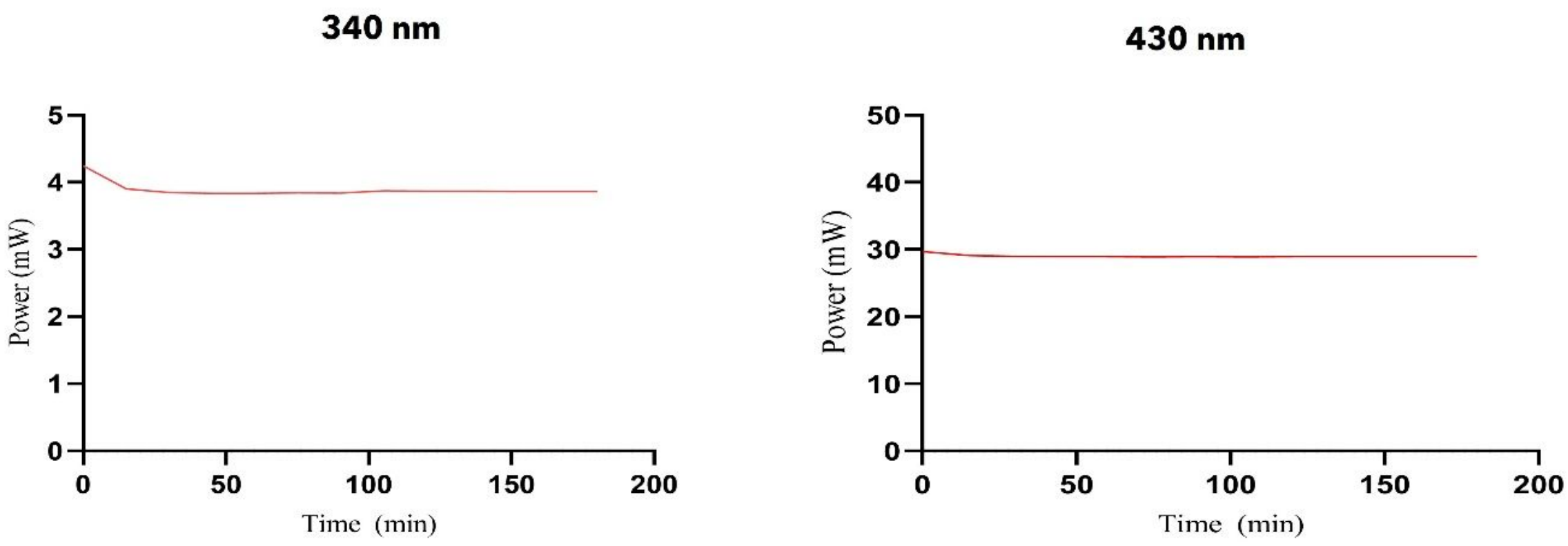

Figure S1. The power stability of the LED source continuously screened for 180 min on 3 consecutive days. The fibre coupled excitation light demonstrated an average power output of 3.94 ± 0.044 mW, 3.88 Mw ± 0.14, 3.87 ± 0.17 mW respectively in the case of 340 nm. On the other hand, 430 nm LED produced 29.07 ± 0.22 mW, 29.15 ± 0,17 mW, 28.80 ± 0,21 mW. In addition, the low-power LED excitation (especially 340 nm), in combination with the CCD-spectrometer minimizes the excitation exposure to few seconds (5-10 seconds). Such reduced exposure can significantly minimize phototoxicity/photodamage. Also, the stable power output ensures the reproducible measurements.

2. Sensitivity of the LED-IAF device

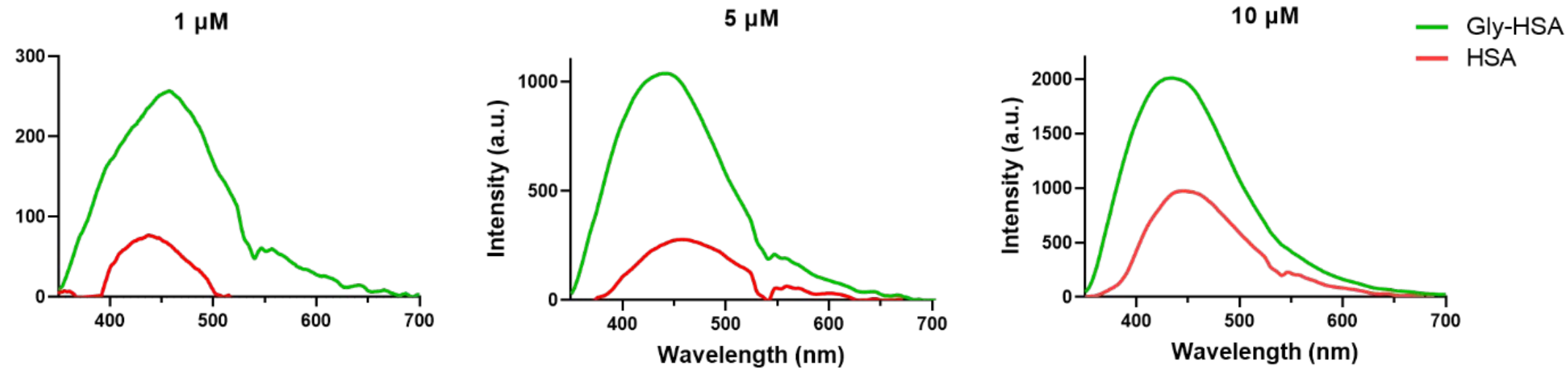

Figure S2. Comparative autofluorescence emission profiles of native and glycated HSA at 340 nm excitation across concentrations of 1-10 µM.

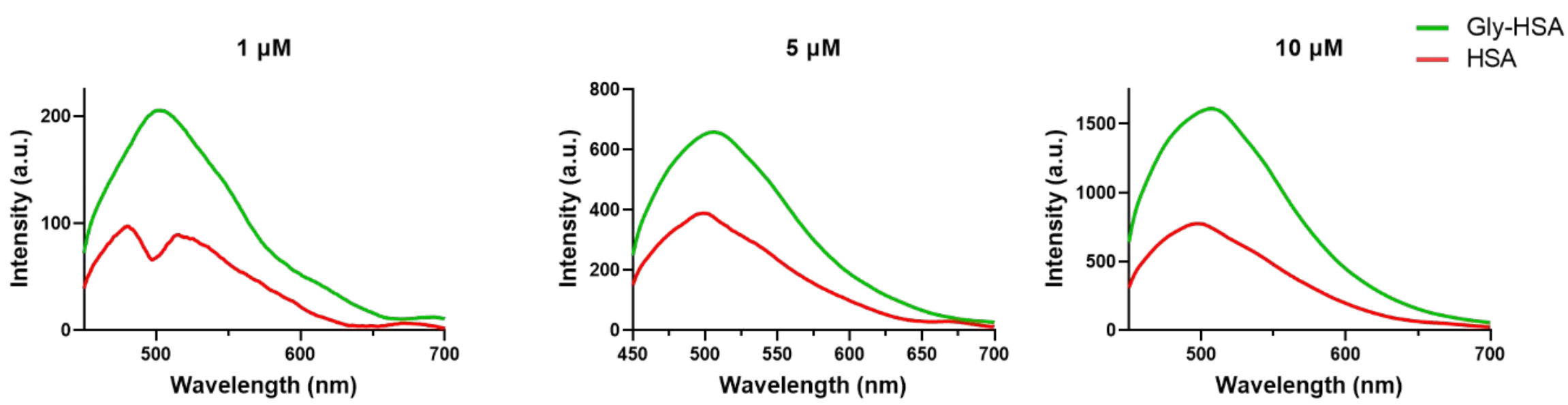

Figure S3. Comparative autofluorescence emission profiles of native and glycated HSA at 430 nm excitation across concentrations of 1-10 µM.

3. Pentosidine and Vesperlysine Reference Spectrum For NN-CLS

A 20 µM solution of L-lysine in phosphate buffer (pH 7.4) was incubated with 40 µM glucose at 37°C for 7 days under dark conditions to generate a vesperlysine fluorescence as reference spectra for NN-CLS analysis [1]. Because pentosidine formation requires the participation of both lysine and arginine residues [2], its formation was absent in this system, enabling the selective production of lysine-derived fluorescent species vesperlysine A&B with an excitation maximum ~440 nm. Also, the reference spectrum is free from other minor AGEs such as argpyrimidine (λ_{em} = 395-400 nm), and other Arg-derived AGEs (λ_{em} = 520-540 nm) because of the absence of arginine residues.

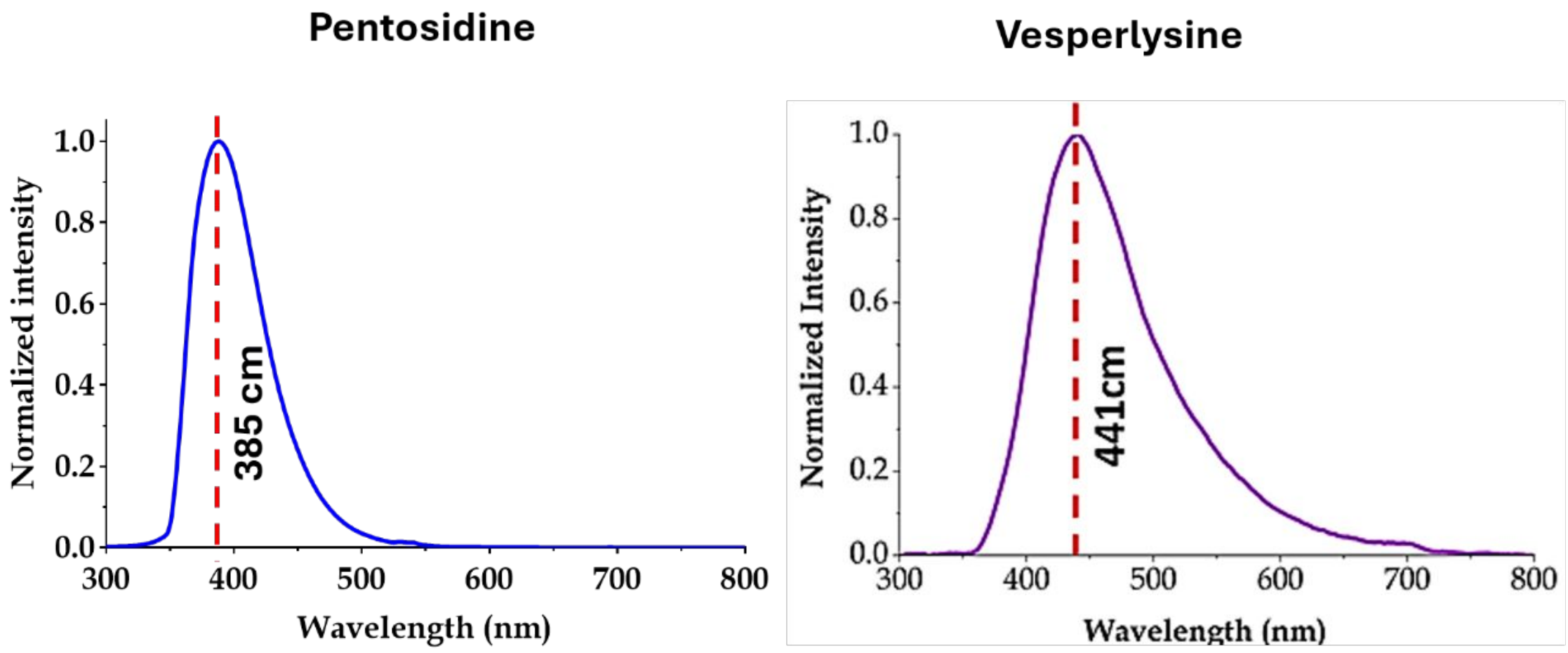

Figure S4: Normalized fluorescence emission spectra of the pentosidine and lysine-derived AGE model (L-lysine + glucose).

4. AGEs specific fluorescence of Elastin

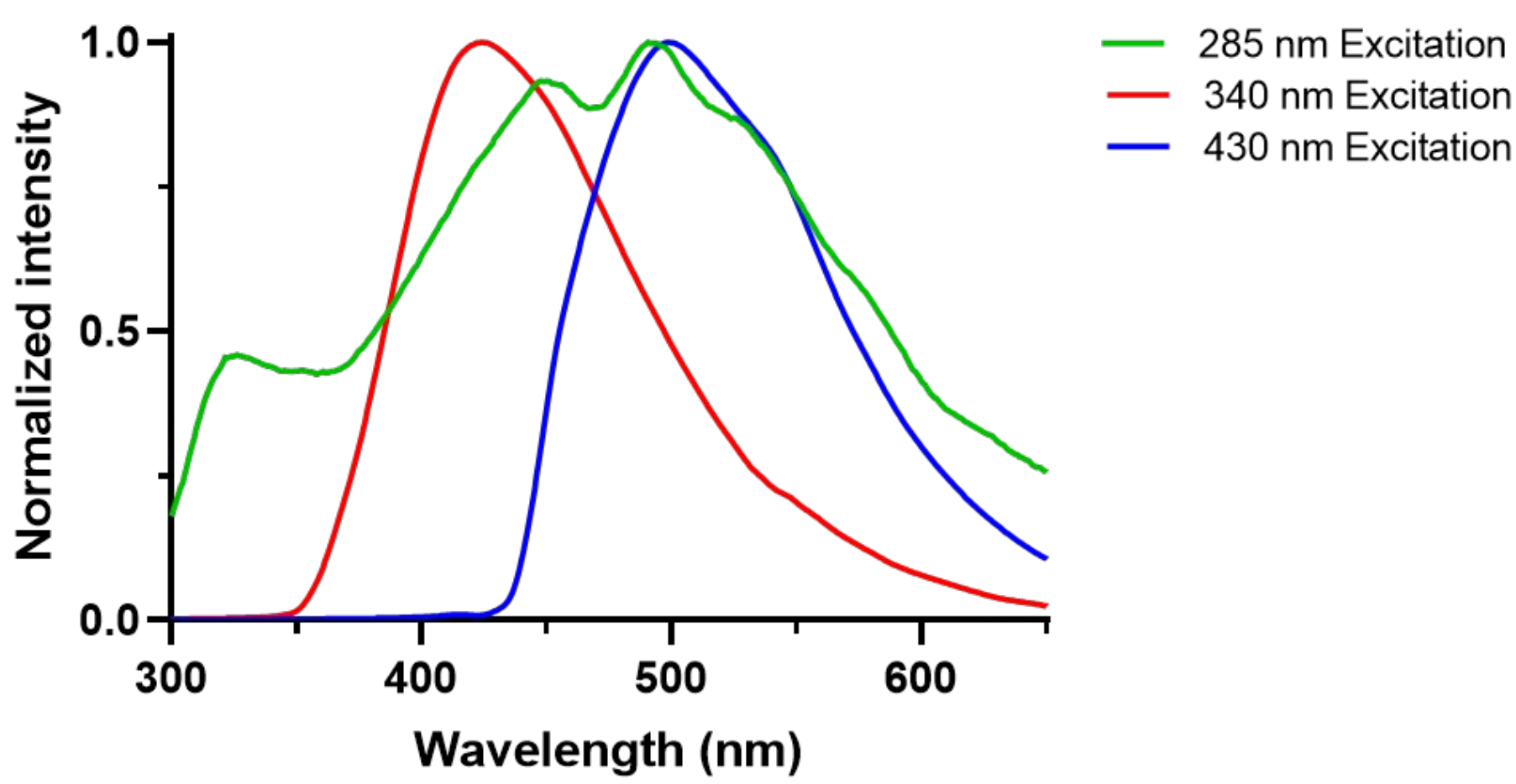

Figure S5. Autofluorescence emission spectra of human elastin at multiple excitation wavelengths, reflecting the accumulation of AGEs over time.

5. Fluorescence decay curve of Native HSA

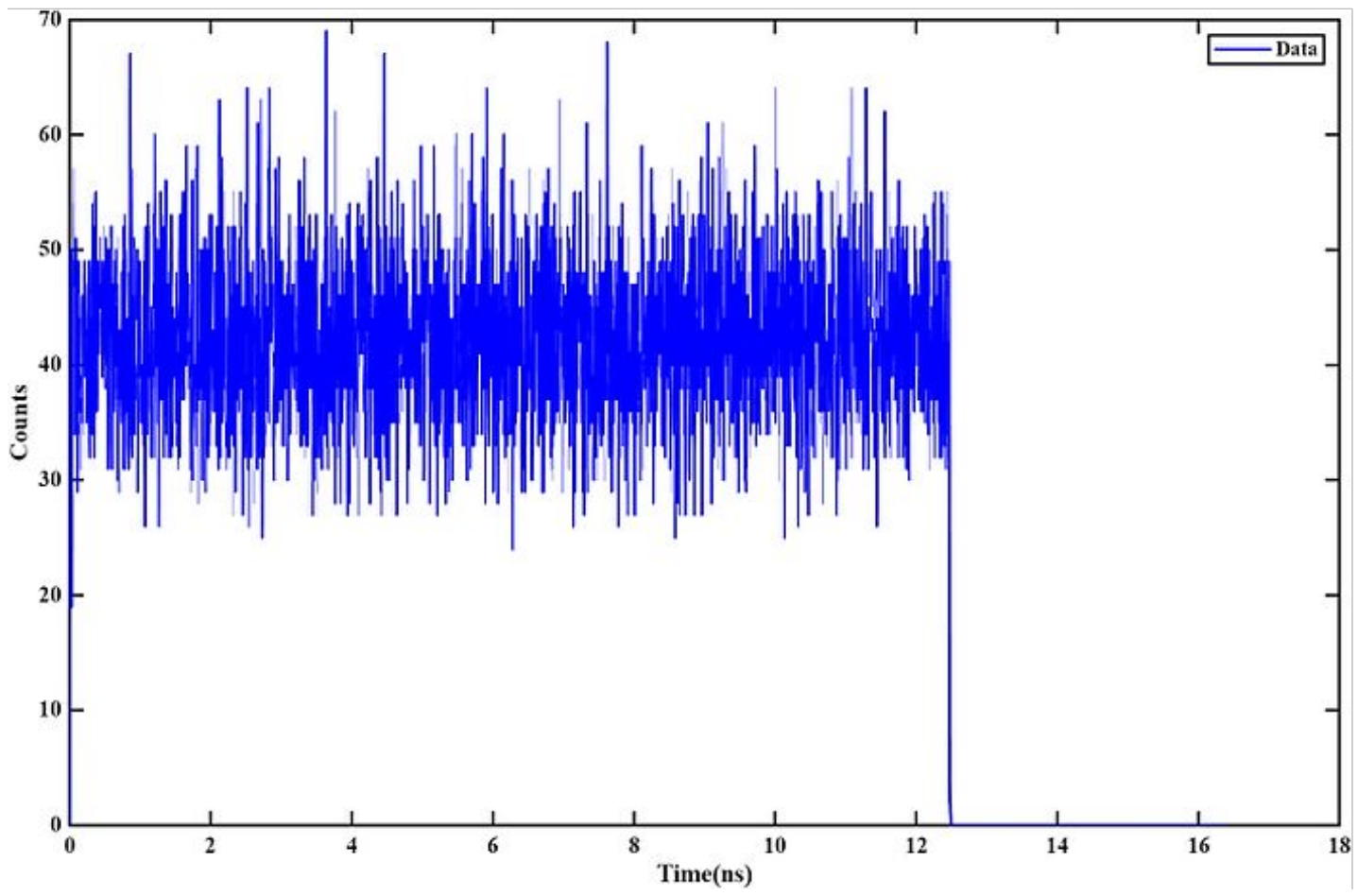

Figure S6. **The representative fluorescence decay to the Native HSA excited at 750 nm 2 photon excitation.** The plot showing a nearly 25-70 photon count distribution across the entire time scale (~0-12 ns). This did not show any characteristic exponential decay. This indicates the absence of fluorescence signals above background noise. The flat profile indicated the recorded data correspond to background or dark counts and not from photons arrival events due to the absorption of the sample.

6. Comparison of AGE detection techniques

Table S1. Comparative overview of analytical techniques used for AGE detection

**Method**	**Sensitivity**	**Selectivity**	**Measurement time**	**Cost**	**Limitations**	**Reference**
HPLC-UV/Vis	< 2 µg/100g protein	Moderate	30-90 mins	Medium	• Limited to UV absorbing AGEs<br>• Poor specificity in complex matrices	3, 4, 5
HPLC-FLD	~0.1 pmol/mg protein (pentosidine); ~1 nM in plasma	Moderate	30-90 mins	Medium	• Detects fluorescent AGEs<br>• Subject to matrix interference	6, 7
LC-MS/MS (HPLC-MS/MS)	~ng-µg range	Very high	30-120 mins	High	• Requires isotope labelled standards; complex operation	8, 9
GC-MS	~0.1-1 nmol/mol lysine	High	45-120 mins	High	• Requires derivatization and extensive sample preparation	4, 9, 10
Skin autofluorescence (SAF/AGE Reader)	No molecular LOD	Low	<5 mins	Low	• Affected by skin conditions and complexion<br>• No specific AGEs identified	11, 12
ELISA	~0.1-5 ng/mL CML	High	3-6 hrs	Medium	• Antibody-cross reactivity	3, 13
Western blot	~ng-µg/lane	Moderate	4-12 hrs	Medium	• Semi-quantitative<br>• Labour intensive<br>• Not suitable for high-throughput	13, 14

Immunohistochemistry (IHC)	No standardized LOD	Moderate	1-2 days	Medium	• Invasive (requires biopsy)	13, 15
NBT-based assay	No standardized LOD	Low	15-60 mins	Very low	• Detects early glycation products only<br>• Prone to interference from other reducing compounds	13, 16
LED-IAF spectroscopy	3.66 picograms/µL pentosidine	Moderate	5 seconds	Low	• Limited to fluorescent AGEs	Current study

7. References

1. Nakamura, K., Nakazawa, Y. & Ienaga, K. Acid-Stable Fluorescent Advanced Glycation End Products: Vesperlysines A, B, and C Are Formed as Crosslinked Products in the Maillard Reaction between Lysine or Proteins with Glucose. *Biochem. Biophys. Res. Commun.* **232**, 227–230 (1997).

2. Schmitt, A., Schmitt, J., Münch, G. & Gasic-Milencovic, J. Characterization of advanced glycation end products for biochemical studies: side chain modifications and fluorescence characteristics. *Anal. Biochem.* **338**, 201–215 (2005).

3. Li, L. *et al.* Advanced Glycation End Products: A Comprehensive Review of Their Detection and Occurrence in Food. *Foods* **12**, 2103 (2023).

4. Patel, S. S. *et al.* Recent Trends in Advanced Glycation End Products in Foods: Formation, Toxicity, and Innovative Strategies for Extraction, Detection, and Inhibition. *Foods* **13**, 4045 (2024).

5. Cengiz, S., Kişmiroğlu, C., Çebi, N., Çatak, J. & Yaman, M. Determination of the most potent precursors of advanced glycation end products (AGEs) in chips, crackers, and breakfast cereals by high performance liquid chromatography (HPLC) using precolumn derivatization with 4-nitro-1,2-phenlenediamine. *Microchemical Journal* **158**, 105170 (2020).

6. Lee, J. S., Chung, Y.-S., Chang, S. Y., Jung, Y.-S. & Kim, S. H. Simple Quantification of Pentosidine in Human Urine and Plasma by High-Performance Liquid Chromatography. *Int. J. Anal. Chem.* **2017**, 1–6 (2017).

7. Scheijen, J. L. J. M., van de Waarenburg, M. P. H., Stehouwer, C. D. A. & Schalkwijk, C. G. Measurement of pentosidine in human plasma protein by a single-column high-performance liquid chromatography method with fluorescence detection. *Journal of Chromatography B* **877**, 610–614 (2009).

8. Wang, D. *et al.* Quantifying carboxymethyl lysine and carboxyethyl lysine in human plasma: clinical insights into aging research using liquid chromatography-tandem mass spectrometry. *BMC Biotechnol.* **24**, 12 (2024).

9. Bao, C. *et al.* Guideline for the Detection of Advanced Glycation End Products. *Food Safety and Health* **4**, 247–258 (2026).

10. Charissou, A., Ait-Ameur, L. & Birlouez-Aragon, I. Evaluation of a gas chromatography/mass spectrometry method for the quantification of carboxymethyllysine in food samples. *J. Chromatogr. A* **1140**, 189–194 (2007).

11. Atzeni, I. M. *et al.* The AGE Reader: A non-invasive method to assess long-term tissue damage. *Methods* **203**, 533–541 (2022).

12. Stirban, A. & Heinemann, L. Skin Autofluorescence – A Non-invasive Measurement for Assessing Cardiovascular Risk and Risk of Diabetes. *Eur. Endocrinol.* **10**, 106 (2014).

13. Ashraf, J. Mohd. *et al.* Recent advances in detection of AGEs: Immunochemical, bioanalytical and biochemical approaches. *IUBMB Life* **67**, 897–913 (2015).

14. Perrone, A., Giovino, A., Benny, J. & Martinelli, F. Advanced Glycation End Products (AGEs): Biochemistry, Signaling, Analytical Methods, and Epigenetic Effects. *Oxid. Med. Cell. Longev.* **2020**, 1–18 (2020).

15. Corica, D. *et al.* Methods to investigate advanced glycation end-product and their application in clinical practice. *Methods* **203**, 90–102 (2022).

16. Lapolla, A., Traldi, P. & Fedele, D. Importance of measuring products of non-enzymatic glycation of proteins. *Clin. Biochem.* **38**, 103–115 (2005).
